# AAA-ATPase FIDGETIN-LIKE 1 and Helicase FANCM Antagonize Meiotic Crossovers by Distinct Mechanisms

**DOI:** 10.1371/journal.pgen.1005369

**Published:** 2015-07-10

**Authors:** Chloe Girard, Liudmila Chelysheva, Sandrine Choinard, Nicole Froger, Nicolas Macaisne, Afef Lehmemdi, Julien Mazel, Wayne Crismani, Raphael Mercier

**Affiliations:** 1 INRA, Institut Jean-Pierre Bourgin, UMR1318, ERL CNRS 3559, Saclay Plant Sciences, RD10, Versailles, France; 2 AgroParisTech, Institut Jean-Pierre Bourgin, UMR 1318, ERL CNRS 3559, Saclay Plant Sciences, RD10, Versailles, France; National Cancer Institute, UNITED STATES

## Abstract

Meiotic crossovers (COs) generate genetic diversity and are critical for the correct completion of meiosis in most species. Their occurrence is tightly constrained but the mechanisms underlying this limitation remain poorly understood. Here we identified the conserved AAA-ATPase FIDGETIN-LIKE-1 (FIGL1) as a negative regulator of meiotic CO formation. We show that *Arabidopsis FIGL1* limits CO formation genome-wide, that *FIGL1* controls dynamics of the two conserved recombinases DMC1 and RAD51 and that *FIGL1* hinders the interaction between homologous chromosomes, suggesting that FIGL1 counteracts DMC1/RAD51-mediated inter-homologue strand invasion to limit CO formation. Further, depleting both FIGL1 and the previously identified anti-CO helicase FANCM synergistically increases crossover frequency. Additionally, we showed that the effect of mutating *FANCM* on recombination is much lower in F1 hybrids contrasting from the phenotype of inbred lines, while *figl1* mutation equally increases crossovers in both contexts. This shows that the modes of action of *FIGL1* and *FANCM* are differently affected by genomic contexts. We propose that FIGL1 and FANCM represent two successive barriers to CO formation, one limiting strand invasion, the other disassembling D-loops to promote SDSA, which when both lifted, leads to a large increase of crossovers, without impairing meiotic progression.

## Author Summary

Sexually reproducing species produce offspring that are genetically unique from one another, despite having the same parents. This uniqueness is created by meiosis, which is a specialized cell division. After meiosis each parent transmits half of their DNA, but each time this occurs, the 'half portion' of DNA transmitted to offspring is different from the previous. The differences are due to resorting the parental chromosomes, but also recombining them. Here we describe a gene—*FIDGETIN-LIKE 1*—which limits the amount of recombination that occurs during meiosis. Previously we identified a gene with a similar function, *FANCM*. FIGL1 and FANCM operate through distinct mechanisms. This discovery will be useful to understand more, from an evolutionary perspective, why recombination is naturally limited. Also this has potentially significant applications for plant breeding which is largely about sampling many 'recombinants' to find individuals that have heritable advantages compared to their parents.

## Introduction

Meiotic crossovers (COs) shuffle parental alleles in the offspring, introducing genetic variety on which selection can act. COs are produced by homologous recombination (HR) that is used to repair the numerous programmed DNA double strand breaks (DSBs) that form in early prophase I. DSBs can be repaired using a homologous template giving rise to COs or non-crossovers (NCOs), or using the sister chromatid leading to inter-sister chromatid exchanges (IS-NCOs or IS-COs). [1]. However, only COs between homologous chromosomes provide the basis for a physical link, forming a structure called a bivalent, and thus COs are required for proper chromosome segregation in most species [2].

DSB formation is catalyzed by the conserved protein, SPO11 [3]. Resection of both sides of the break produces two 3′ single strand overhangs. One of these overhangs can invade a homologous template, either the homologous chromosome or the sister chromatid, producing a joint DNA molecule, the displacement loop (D-loop) [4]. Two strand-exchange enzymes catalyze this template invasion step: RAD51 and the meiosis-specific DMC1 polymerize on the single-strand DNA and promote invasion of the intact homologous template [5,6]. The choice of the template for repair is crucial to form COs during meiosis, and the respective roles of DMC1, RAD51 and their co-factors in ensuring inter-homologue bias and avoiding inter-sister repair remains to be fully understood [6–10]. Studies in several organisms have demonstrated that multiple co-operative factors influence meiotic template choice [11]. In budding yeast it has been shown that while both DMC1 and RAD51 are recruited at DSB sites, RAD51 strand-exchange activity is not required for strand invasion at meiosis, and that RAD51 is relegated to a role as a DMC1 co-factor [6]. The same is likely true in *Arabidopsis* [10]. In plants, an additional player, the cyclin SDS, is essential for DMC1 focus formation, DMC1-mediated bias toward inter-homolog DSB repair and CO formation [12,13].

Following D-loop formation, the invading strand then primes DNA synthesis, using the complementary strand of the invaded duplex as a template. The mode of repair of this joint molecule determines the outcome as a CO or an NCO. First, the extended invading strand can be unwound and can re-anneal with the second end of the DSB, a mechanism called SDSA (synthesis-dependent strand annealing), leading to the repair of the breaks exclusively as NCOs [14]. Alternatively two pathways that produce COs co-exist in many species including *Arabidopsis* [15,16]: the first depends on a group of proteins collectively referred to as the ZMM proteins [17] and the MLH1-MLH3 proteins (class I CO), which promotes the formation of double Holliday junctions and their resolution as COs [18]. The second CO pathway, that can produce both COs and NCOs, depends on structure-specific endonucleases including MUS81 (class II COs) [18]. Class I COs are sensitive to interference: they tend to be distributed further apart—from one another—along the same chromosome than expected by chance. In contrast, class II COs are distributed independently from each other [19], but not completely independently from class I COs as recently shown in tomato [20]. In *Arabidopsis*, the ZMM pathway accounts for the formation of about 85% of COs, the class II pathway being minor [21,22].

Despite an excess of recombination precursors, most species only form close to the one, obligatory, CO per chromosome [23]. Mechanisms underlying this limitation are currently being unraveled, but still very few anti-CO proteins are known [24–30]. The helicase FANCM, with its two co-factors MHF1 and MHF2, defined the first known anti-CO pathway in plants and limit class II COs [24,31].

In this study, continuing the genetic screen that identified *FANCM* and *MHF1-MHF2*, we identify *FIDGETIN-Like-1* (*FIGL1*) as a new gene limiting meiotic CO formation. Human FIGL1 was previously shown to interact directly with RAD51 and to be required for efficient HR-mediated DNA repair in human U2OS cells [32]. Here we show that *FIGL1* limits class II COs at meiosis and that *FANCM* and *FIGL1* act through distinct mechanisms to limit meiotic crossovers. While FANCM likely unwinds post-invasion intermediates to produce NCOs [24,26], we provide evidence that FIGL1 limits meiotic CO formation by regulating the invasion step of meiotic homologous recombination.

## Results

### A genetic screen for suppression of the lack of chiasmata in *zmm* mutants identified FIGL1

CO-deficient mutants (e.g. *zmm* mutants) of *Arabidopsis* display reduced fertility, noticeable by their reduction in fruit length, due to homologous chromosomes not segregating correctly at meiosis I and the ensuing formation of aneuploid gametes. We designed a genetic screen to identify anti-CO factors in *Arabidopsis*, as described previously [24]. Using fruit length as a proxy for the level of CO formation, we screened for suppressors of CO-deficient mutants (the *zmm* mutants; *zip4*, *shoc1*, *hei10*, *msh4* and *msh5*, S1 Table), based on the idea that mutation of ‘anti-CO’ genes would restore the level of CO formation and therefore correct chromosome segregation and fertility of the plants. It should be noted that this screen would be unable to recover mutants with elevated class I COs only, but could recover mutants in which class II COs or both CO classes are increased.

**Fig 1:**
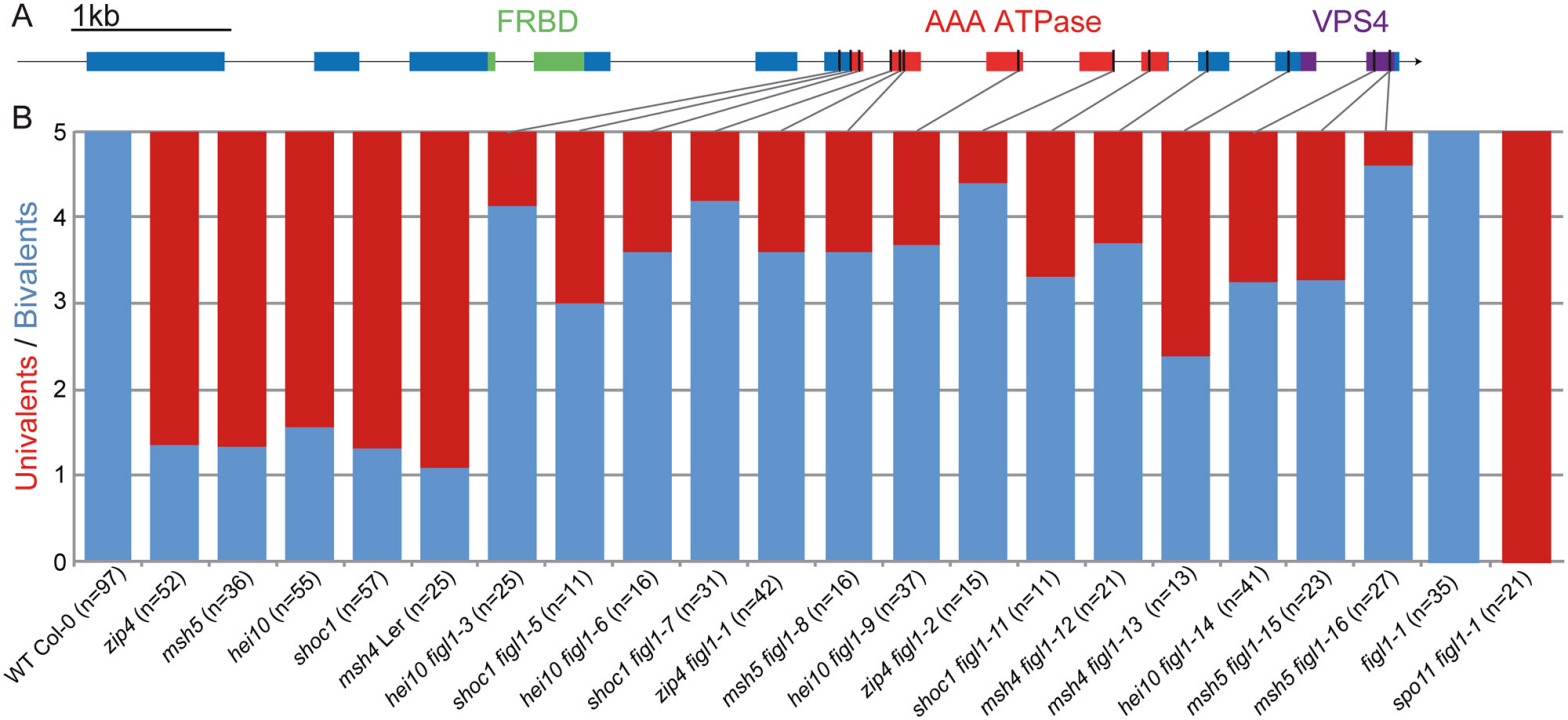
Mutations in *FIGL1* restores bivalent formation in *zmm* mutants. A: Gene model of the *FIDGETIN-Like-1* gene, exons appear as blue boxes, the conserved domains are indicated in green, red and purple. Black lines represent the position of the point mutations. B: Univalent pairs (red) and bivalents (blue) count of metaphase I male meiocytes in wild type, *zmm* mutants (*zip4*, *msh4*, *msh5*, *hei10* and *shoc1*) and in *zmm figl1* double mutants, as well as *figl1* single mutants and *figl1-1 spo11* double mutants. All genotypes are in a Columbia-0 background, except for *msh4 figl1-12* and *msh4 figl1-13* which are in a Landsberg erecta background (Ler).

The *zip4* suppressor screen led to the isolation of three complementation groups. The study of the first two revealed FANCM and MHF1-MHF2 as anti-CO proteins that act in the same pathway [24,31]. Here we focus on the third complementation group that has two allelic suppressors, *zip4(s)4* and *zip4(s)5* (S1 Table). Using mapping and whole genome sequencing, we identified a putative causal mutation in *zip4(s)5*, a deletion of one base pair in the gene *At3g27120*. The allelic suppressor *zip4(s)4* contained also a mutation in this gene, showing that the *At3g27120* mutation is responsible for the fertility restoration. This was further consolidated by the identification of 12 other allelic mutations in the other *zmm* screens (Fig 1 and S1 Table). In wild-type *Arabidopsis* meiosis, the five pairs of homologs always form five bivalents at metaphase I, whereas *zmm* mutants have few bivalents (~1.3 bivalent per meiosis, Fig 1B). All *figl1* alleles largely, but never entirely, restored bivalent formation in all *zmm* backgrounds tested (Fig 1B). No growth or development defects were observed in these mutants.

Sequencing of the cDNA revealed a mis-annotation as the two *in silico* predicted genes *AT3G27120* and *AT3G27130* correspond to one mRNA *in vivo* (Genbank accession KM055500; S1A Fig). Reciprocal BLAST analysis showed that the protein encoded by this gene is the single representative of the AAA-ATPase FIDGETIN family in *Arabidopsis* (S1B Fig). The FIDGETIN protein family comprises three proteins in mammals (FIDGETIN, FIDGETIN-Like-1 and FIDGETIN-Like-2). Phylogenetic analysis showed that only *FIDGETIN-Like-1* (*FIGL1*) is conserved in other branches of eukaryotes, including *Arabidopsis* (S1B and S1C Fig). *FIGL1* is present in most eukaryotic clades; however, we could not detect any representative of the *FIDGETIN* family in fungi, with the exception of the early divergent *Microsporidia* genera that possesses a *FIGL1*, suggesting that this gene was lost early after the fungi lineage divergence. Mouse FIGL1 is highly expressed in meiocytes [33], and human FIGL1 has been reported to be essential for efficient HR-mediated DNA repair in somatic cells, through a direct interaction with RAD51 [32]. FIGL1 also interacts with KIAA0146/SPIDR which is involved in HR and that in turns interacts with RAD51 and BLM, the latter is a helicase involved in DSB repair known to antagonize crossover formation [34]. All these findings point towards a conserved role for FIGL1 in homologous recombination.

Attempts to localize the FIGL1 protein *in planta*, and notably in meiocytes, were unsuccessful. However, using over-expression of the protein in Tobacco leaves, we were able to detect a strong signal in the nucleus (S1D Fig), suggesting that FIGL1 is targeted to the nucleus, at least when over-expressed in somatic cells.

### *figl1* increases meiotic recombination in a multiplicative manner with *fancm*

**Fig 2:**
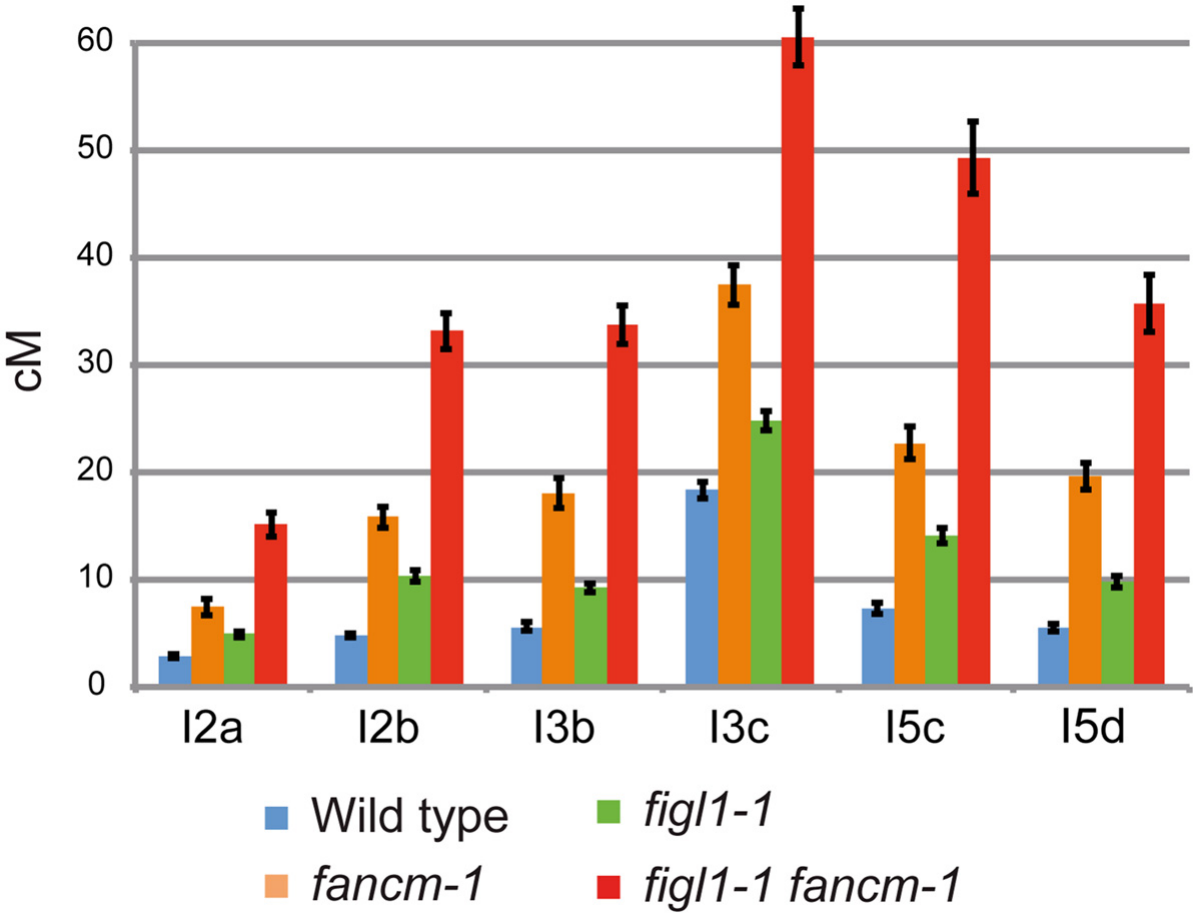
FIGL1 limits meiotic CO independently of FANCM. Genetic distances (in cM) measured from tetrad analysis in a series of intervals across *Arabidopsis* genome: I2a and I2b are adjacent intervals on chromosome 2 and so on for the other couples of intervals. Error bars: SD. On all intervals all genotypes are significantly different from each other (Z-test, p<0.01).

To directly test the effect of *FIGL1* mutation on CO frequency, we performed tetrad analysis to measure recombination in a series of intervals defined by markers conferring fluorescence in pollen grains (Fluorescent-Tagged Lines—FTLs) [35] (Fig 2 and S2 Fig). These data showed that: (i) the *figl1-1* mutation restores recombination of the *zip4* mutant, in accordance with the restoration of bivalent formation (S2A Fig); (ii) in the single *figl1-1* mutant, CO frequency is increased in each of the six intervals tested (Z-test, p<10^−6^), on average by 72% compared to wild type, demonstrating that *FIGL1* is a barrier to CO formation also in wild type (Fig 2); (iii) while single *fancm-1* mutants display a three-fold increase in genetic distances on average (p<10^−6^ and [24]), a six-fold increase is observed in the *figl1-1 fancm-1* double mutant compared to wild type (p<10^−6^) on average on the six intervals tested, which is higher than either single mutant (p<10^−6^), showing that the effects of these mutations are multiplicative (Fig 2). This result shows that FIGL1 and FANCM act by two distinct mechanisms to limit crossover formation at meiosis. The net effect being multiplicative rather than additive further suggests that FIGL1 and FANCM act sequentially or synergistically at the same step to limit the flux of recombination intermediates toward CO formation.

The *figl1-1 fancm-1* plants are indistinguishable from wild type in terms of growth and fertility (57.5±6 seeds per fruit in *figl1-1 fancm-1* (n = 13) and 55.2±6 in wild type (n = 24; T-Test p = 0.42)). Meiosis proceeds normally in this double mutant leading to the conclusion that a large increase in CO frequency does not cause any dramatic defects in chromosome segregation.

### Genetic maps of *figl1* reveal a marked increase in CO formation in distal regions of chromosomes

**Fig 3:**
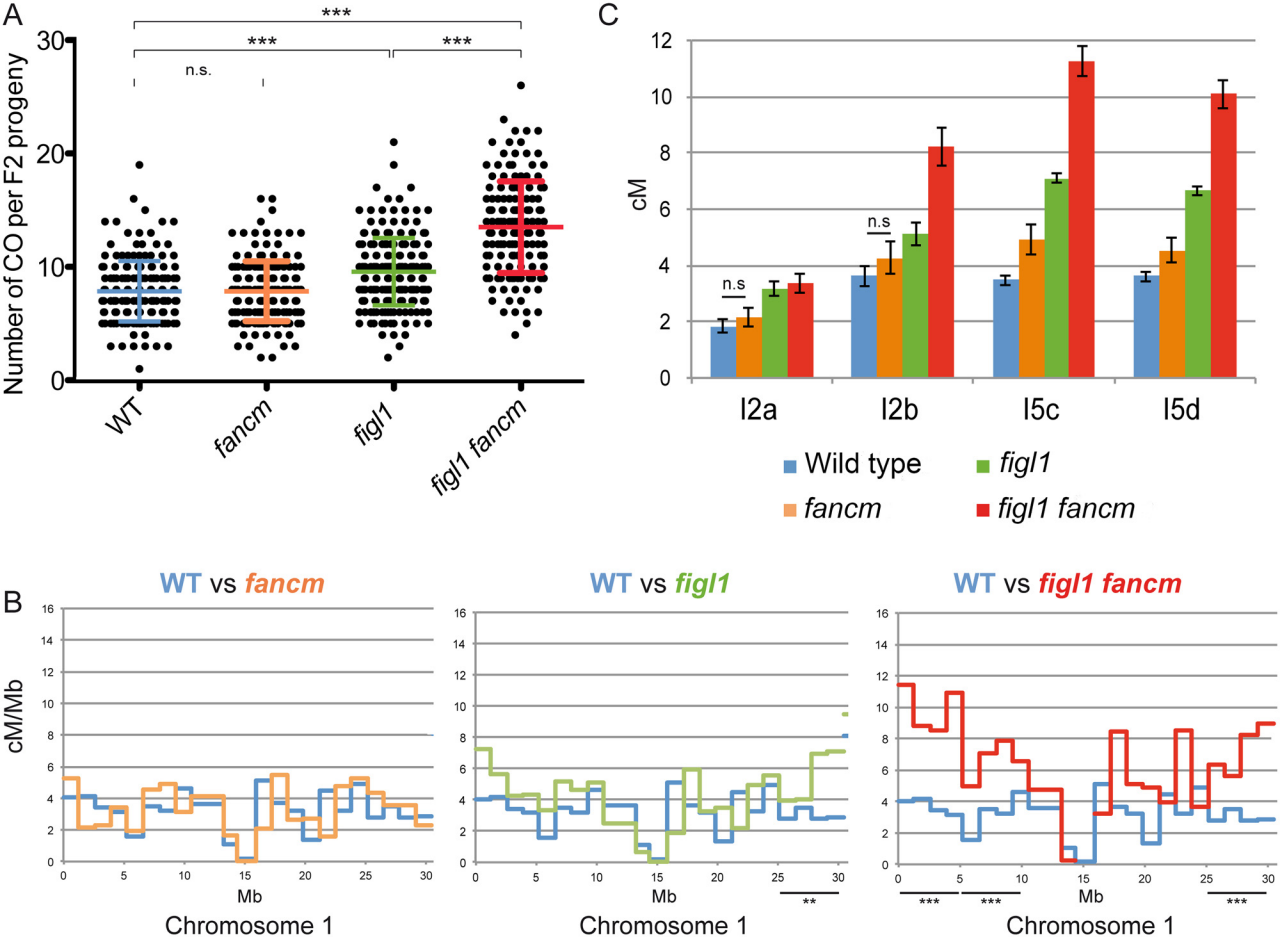
The effect of *figl1* and *fancm* on recombination in hybrids. A: CO count in each F2 progeny obtained from parent plants from Columbia-0/Landsberg (Col/Ler) F1 hybrids. Means and SD are indicated. n.s.: not significant; *** indicates significant difference, T-Test p<0,001. B: Recombination frequency (in cM/Mb) along chromosome I compared to wild type for each genotype. Difference in recombination frequency was tested along the genome on ~5Mb intervals (see methods). See also S5A Fig. C: Genetic distances (in cM) measured from tetrad analysis in a series of intervals, in Col/Ler F1 hybrids. All genotypes on all intervals are significantly different from wild type (Z-test, p<0.03), except when noted (n.s.: not significant).

We analyzed the genome wide frequency and CO distribution using segregation of polymorphisms between different strains. While all alleles described above were identified in the Columbia-0 (Col-0) strain, we obtained mutant alleles in another genetic background by performing a suppressor screen of *msh4* in another strain, Landsberg erecta (Ler) (S1 Table). The Ler *figl1-12* allele displayed the same ability to restore bivalents of *zmm* mutants as its Col-0 counterparts (Fig 1). Genetic maps were obtained through segregation analysis of 91 markers (S2 Table) on F2 plants obtained by self-fertilization of *figl1* (*figl1-1/figl-12*) and wild-type Col-0/Ler F1s. This showed a global increase of COs genome wide, with a 25% increase of observed crossover number per F2 plant in *figl1* compared to wild type (Fig 3A; T-Test p<0,001). The increase is variable along chromosomes, with a more marked increase in the distal regions than close to centromeres: all ~5Mb intervals that individually show a significant increase compared to wild type are sub-telomeric (Fig 3B and S3 Fig). Conversely, the intervals spanning the centromeres, which have a low recombination frequency in wild type, remain similarly low in *figl1*.

In addition, tetrad analyses were performed on F1 Col-0/Ler hybrid plants using FTLs. In the hybrid *figl1* mutant, we observed a 79% average increase in CO frequency, on the four intervals tested, compared to the sister wild type controls (Fig 3C). This increase is similar to the one observed in the inbred Col-0 background on the same intervals, and to the observed increase with marker segregation analysis on the same region (S3 Fig). These increases are higher than the average increase genome wide (25%), likely because the FTL intervals used are positioned rather distally on the chromosomes. These genetic data confirm that *FIGL1* is a barrier to CO formation in wild-type inbreds and hybrids.

### *FANCM* mutation increases crossovers efficiently in inbreds but minimally in hybrids

The *msh4* screen in a Ler background also led to the identification of several *fancm* mutants with a large increase in bivalent formation, including *fancm-10* (S1 Table). Bivalent frequency in *fancm-10 msh4* (Ler) was as high as in *fancm-1 msh4* (Col-0) (S4A Fig), confirming that *fancm* is a *bona fide* suppressor of *zmm* in both Columbia and Landsberg backgrounds. As described above for *figl1*, we performed marker segregation analysis using the same set of 91 markers in F2 populations derived by self-pollination of *fancm* F1 hybrids. In contrast to the *fancm* inbred, the observed number of COs in *fancm* hybrids Col-0/Ler was the same as in the hybrid wild type (7.8 COs per cell; Fig 3A). The observation of CO distribution (Fig 3B and S3 Fig) did not reveal differences between *fancm* and wild type. Tetrad analysis recapitulated this observation with an average 200% increase when *fancm* is compared to wild type in Col-0 (this study and [24]) but only an average 22% increase in the Col-0/Ler F1s on the four intervals tested (ranging from no detected increase to a significant 42% increase p<10^−8^, Fig 3C). This suggests the anti-CO activity of *FANCM*, which is large in inbreds, is strongly diminished in hybrids.

Further lines of evidence support this conclusion. First, marker segregation analysis in a pure Col-0 background confirmed a strong effect of *fancm* in increasing COs (S4B Fig). Second, while *fancm* very efficiently restores bivalent formation of *zmm* mutants in Col-0, Ler, or Wassilewskija (Ws) inbred strains, it is not the case in both Col-0/Ler and Col-0/Ws F1 hybrids (S4A Fig). Finally, an independent study [36] showed that the effect of *fancm-1* on increasing CO is also abolished in an F2 Col-0/Catania hybrid: when the tested interval was heterozygous Col-0/Cat, *fancm-1* had no effect on CO frequencies in this experiment. These data confirm that the effect of *fancm* on increasing COs is strongly diminished in hybrid contexts.

### *figl1* and *fancm* have multiplicative effects on CO in hybrids

Tetrad analysis showed that the mutation of both *FIGL1* and *FANCM* in a Col-0/Ler F1 led to an increase of CO frequency compared to wild type on the four intervals tested (Fig 3C), with a 2.5-fold increase on average. This is higher than either single mutant (1.8 and 1.2, respectively), showing that *figl1* and *fancm* have multiplicative effects also in Col/Ler F1s. However, this increase is lower than what was observed when comparing *figl1 fancm* and wild type in inbred Col-0 strains (6 fold). This is likely due to *fancm* having a lesser increase in CO frequency in hybrids than in inbreds. Indeed there is the same effect of mutating *figl1* in a *fancm* mutant either in hybrid or inbred (*figl1 fancm vs*. *fancm*: 1.96 and 2.03 average ratio, in Col-0/Ler and Col-0 respectively) whereas mutating *fancm* in *figl1* mutant is much less effective in the hybrid than in the Col-0 inbred (*figl1 fancm vs*. *figl1*: 1.42 and 3.45 average ratio, in Col-0/Ler and Col-0 respectively). In the genome wide analysis, the observed number of COs per plant (Fig 3A) increased from 7.8 in WT to 13.5 in *figl1 fancm* (T-Test, p<10^−4^), which is higher than both single mutants (7.8 in *fancm* and 9.6 in *figl1*, T-Test, p<10^−4^). While we detected no effect of *fancm* on the number of COs genome-wide in the wild-type background, *fancm* had a significant effect in the *figl1* background (13.5 *vs*. 9.6 COs, p<10^−4^). The increase in COs in *figl1 fancm* is significant in the distal regions, and not detectable close to centromeres (Fig 3B and S3 Fig). Increased COs close to centromeres have been reported to be associated with chromosome mis-segregation in budding yeast and humans [37–39]. We did not observe segregation defects in *figl1 fancm*, suggesting that only proximal extra-COs are detrimental for correct chromosome segregation. Altogether, these data showed that (i) FANCM is a more important anti-CO protein in Col-0 than in the hybrid, contrary to (ii) FIGL1 which is equally efficient in both contexts; (iii) FIGL1 and FANCM have multiplicative effects on limiting COs in F1 hybrids, as in inbreds.

### *FIGL1* antagonizes *MUS81*-dependent crossover formation

**Fig 4:**
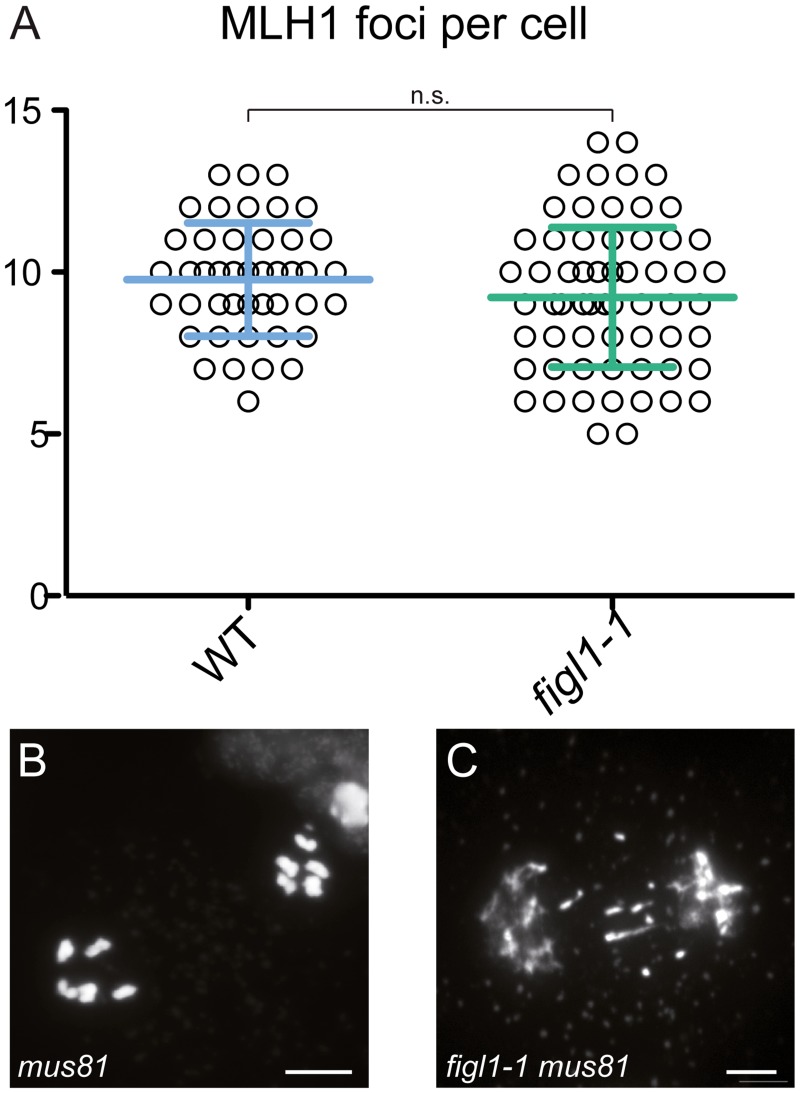
*FIGL1* limits *MUS81*-dependent CO formation. A: MLH1 foci number is unchanged in *figl1-1* compared to wild type. B-C: Anaphase I in *mus81* (B) and *figl1-1 mus81* double mutant (C), the latter displays chromosome fragments indicative of unrepaired recombination intermediates. Scale bar = 5μm.

We then investigated the origin of the *figl1* extra-COs. Mutating *SPO11-1* in *figl1-1* abolished bivalent formation (Fig 1), showing that CO formation in *figl1-1* arises from SPO11-dependent DSBs. Two classes of COs coexist in *Arabidopsis*: one dependent on ZMM proteins, marked by the MLH1 protein and subject to interference; and one involving the endonuclease MUS81 and insensitive to interference [22,40,41]. Immuno-labeling of MLH1, which specifically marks designated sites of class I COs, did not reveal any differences between *figl1-1* and wild type (Fig 4) suggesting that the extra-COs observed in *figl1-1* are not class I crossovers. Corroborating this, the strength of interference measured genetically was weaker in *figl1-1* compared to WT on all intervals tested (S2D Fig), suggesting that extra-COs in *figl1* are not sensitive to interference. Moreover, in the *figl1-1 mus81* double mutant, entangled meiotic chromosomes and sterility were observed (Fig 4). This is not observed in either single mutant, showing that *MUS81* becomes essential for the proper repair of recombination intermediates in *figl1-1*. We thus propose that FIGL1, similar to FANCM [24], prevents the formation or the persistence of intermediates that require MUS81 for repair, and whose resolution leads to extra-CO formation (without affecting the number of class I COs). Contrary to *fancm* however, no growth or developmental defect was observed when *FIGL1* was mutated in a *mus81* background, indicating that the role of *FIGL1* in antagonizing the MUS81 pathway may be specific to meiosis. The additional meiotic COs produced in the absence of FIGL1 are likely dependent on MUS81, but we cannot exclude that other—unidentified—activities contribute to the formation of these COs.

### Synaptonemal complex length is not affected in *fancm* or *figl1*

**Fig 5:**
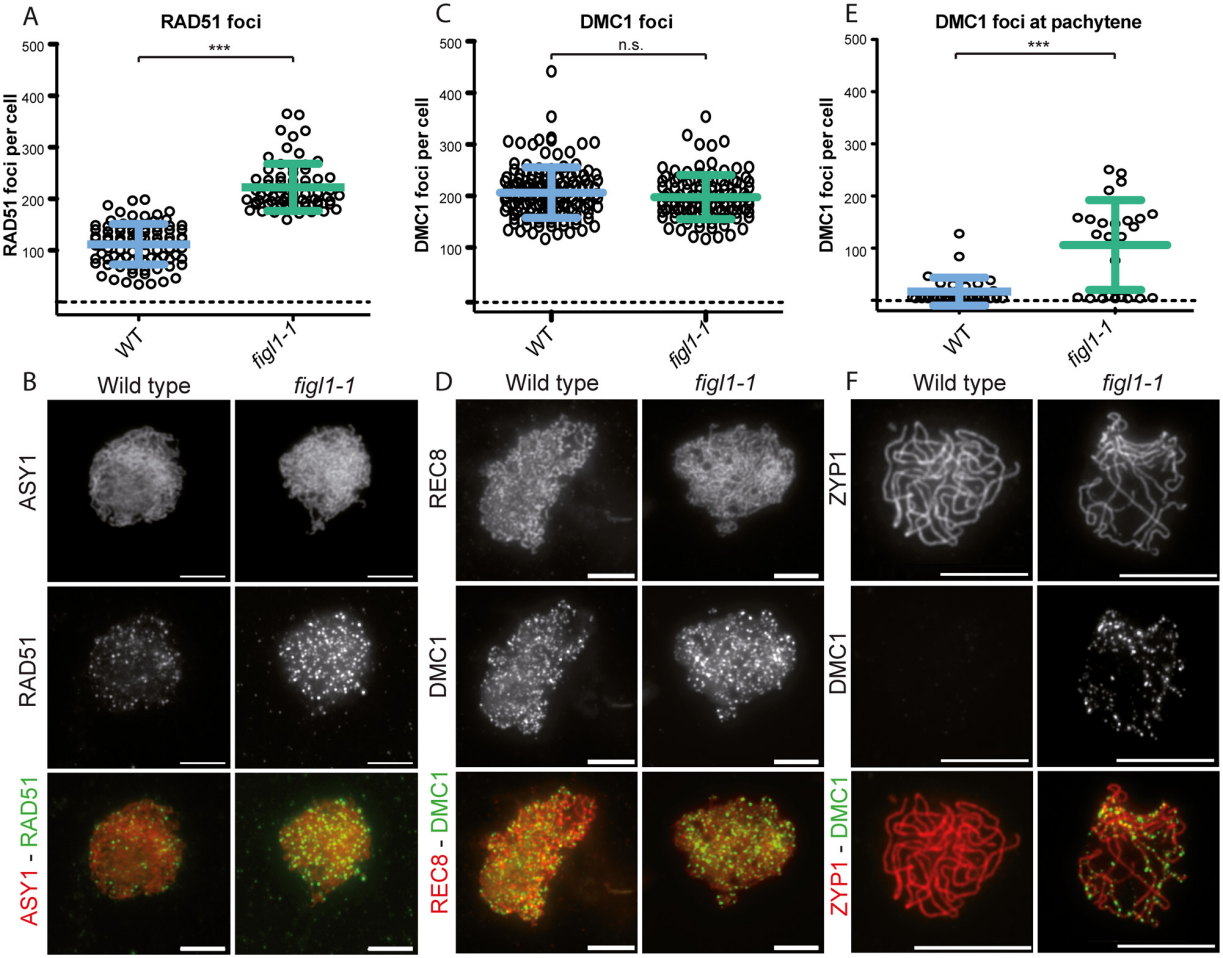
The dynamics of DMC1 and RAD51 are modified in *figl1*. A and C: Number of RAD51 and DMC1 (respectively) foci count per positive cell throughout prophase in both wild type and *figl1-1* mutant. n.s.: not significant; *** T-test p<0,001. B: Illustration of RAD51immuno-localization at leptotene in wild type and *figl1-1* mutant, with the axis protein ASY1used as a counterstain. D: Illustration of DMC1immuno-localization at leptotene in wild type and *figl1-1* mutant, with the REC8 cohesin used as a counterstain. The same exposure and treatment parameters have been applied to all images of both wild type and *figl1-1*. E: DMC1 foci count in pachytene cells (*** T-test p<0,001). ZYP1 staining was used as a marker for full synapsis, indicative of the pachytene stage. F: Illustration of DMC1 immuno-localization at pachytene, with ZYP1 as a counterstain.

**Fig 6:**
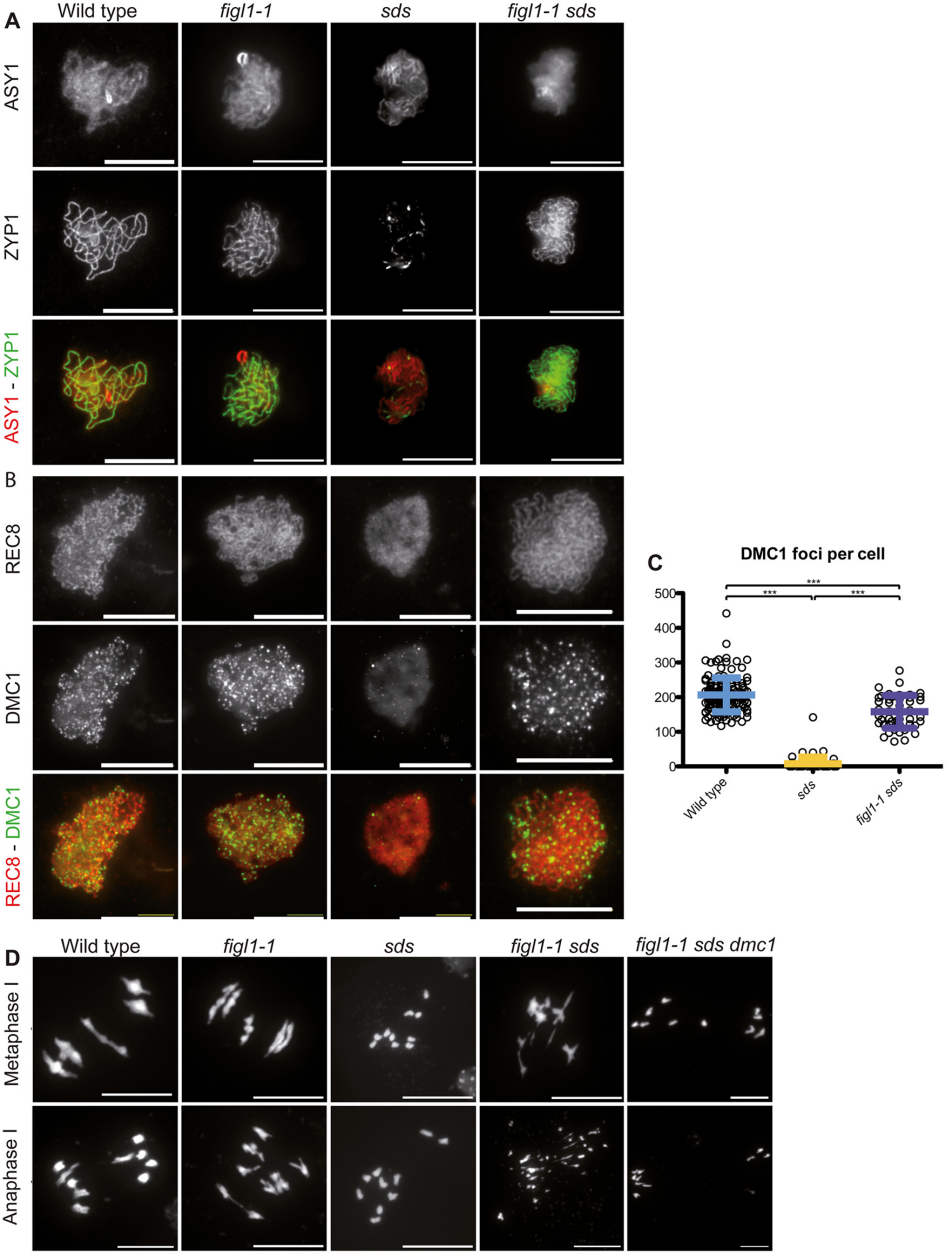
*FIGL1* genetically interacts with *SDS*. A: ZYP1 immuno-localization as a marker of synapsis, with the chromosome axis protein ASY1 used as a counterstain, showing that synapsis is restored in *figl1-1 sds* double mutant compared to *sds* single mutant. B: DMC1 immuno-localization with the REC8 cohesin used as a counterstain showing that DMC1 foci formation is restored in *figl1-1 sds* compared to *sds*. C: Quantification. D: DAPI staining of meiotic chromosome spreads at metaphase I (top) and anaphase I (bottom). While *sds* mutant meiosis displays 10 univalents, *figl1-1 sds* meiosis presents bivalent like structures at metaphase I and chromosome fragments at anaphase I, indicative of unrepaired recombination intermediates. Bivalent and fragmentation are not observed in *figl1-1 sds dmc1*.

Synapsis, the intimate association of homologous chromosomes along their entire length observed at pachytene, was not different in *figl1-1*, *fancm* and wild type, as observed by immuno-localization of the axial element and transverse filament of the synaptonemal complex (SC), ASY1 and ZYP1 respectively (Figs 5 and 6 and S5 Fig). ZYP1-marked SC length in both mutants was not different from wild type (*figl1* 113.4 μm [n = 4] and *fancm* 125.6 μm [n = 32], *vs*. 125.5 μm [n = 33] in wild type). This shows that largely increasing the frequency of non-interfering COs does not affect the SC length. SC length has been shown to be longer in male than in female *Arabidopsis* meiosis, and the male genetic map length is also greater in male than in female [42,43]. Such correlated variations in SC length and CO number were also reported between male and female in various species, among individuals in the same species, and among meiocytes in a single organism (discussed in [42]). If CO number and SC length are linked, one attractive hypothesis would be that these fluctuations may only depend upon the ZMM COs, while increasing class II COs would have no effect.

### *FIGL1* regulates RAD51 and DMC1 foci dynamics

We performed co-immuno-localization experiments of the axis proteins ASY1 and RAD51, as well as the REC8 cohesin and DMC1, in both wild type and *figl1* (Fig 5). In wild-type leptotene/zygotene cells we observed a mean number of 206 DMC1 foci and 111 RAD51 foci (Fig 5). In *figl1* sister plants, a sharp two-fold increase of the number of RAD51 foci was observed (p<10^−3^), while the number of DMC1 foci was unchanged (p = 0.14). This shows that FIGL1 limits the number of RAD51 foci, but not of DMC1 foci in wild type. The increase of RAD51 foci number could suggest that the number of DSBs is increased in *figl1* compared to wild type, but the absence of increase of DMC1 foci number argues against this interpretation. We thus favor the interpretation that the dynamics of RAD51 foci are modified, either being associated with a higher proportion of DSBs or/and persisting longer on chromosomes. We then performed double immuno-localization of RAD51 and DMC1 in wild type and *figl1* (S6 Fig). In wild type, all DMC1 positive cells were also positive for RAD51 foci (n = 17), while only 36% RAD51-positive cells were also positive for DMC1 foci (n = 59). This suggests that, in wild type, DMC1 is present as foci on chromosomes in a shorter period than RAD51. In *figl1*, like in wild type, all DMC1 positive cells were positive for RAD51 foci (n = 40), however 95% of RAD51-positive cells were also showing DMC1 foci (n = 63). In addition, co-immuno-localization of ZYP1 and DMC1 showed that DMC1 foci persisted at pachytene cells in *figl1* but not in wild type (Fig 5E and 5F). Thus, the dynamics of DMC1 foci with respect to RAD51 and synapsis appears to be modified in *figl1* with a longer window of presence.

The plant-specific cyclin *SDS* is required for DMC1 focus formation/stabilization [12,13]. While DMC1 focus formation is virtually abolished in *sds*, in *figl1-1 sds* the formation of DMC1 foci was restored to ~70% of wild-type level (Fig 6A and 6B). This shows that *FIGL* limits DMC1 foci formation in *sds* or accelerates turnover of DMC1 complexes in *sds*, and that SDS promotes DMC1 foci formation in both wild type and *figl1*. Thus *SDS* and *FIGL1* have antagonistic, direct or indirect, roles toward DMC1 foci formation.

Synapsis is strictly dependent on DSB formation and inter-homolog strand invasion in *Arabidopsis* [16,44]. Accordingly, no synapsis is observed in the absence of either of the strand exchange promoting proteins DMC1 or RAD51. Similarly, no synapsis is observed in *sds* suggesting that inter-homolog strand invasion is also abolished in this mutant [12,13,45–47]. In contrast, synapsis was restored in *figl1 sds* (Fig 6C and S7 Fig), showing that FIGL1 prevents synapsis, and thus presumably inter-homolog strand-invasion, in *sds*. No synapsis was observed in *figl1 dmc1*, *figl1 rad51*, *figl1 sds dmc1*, or *figl1 sds rad51* (S7 Fig). DMC1 and RAD51 are thus essential for synapsis in all contexts. This suggests that FIGL1 limits RAD51/DMC1 mediated inter-homolog strand invasion, which is antagonistic to the function of SDS. Mutation of *FANCM* did not restore synapsis or bivalent formation in *sds* (S7 Fig), confirming that FIGL1 and FANCM act through distinct mechanisms.

In *rad51*, massive chromosome fragmentation occurs at metaphase/anaphase I, indicative of failed DSB repair. In contrast DSB repair is efficient in *dmc1* and *sds*, presumably using the sister chromatid as a template. This repair is *RAD51* dependent, as fragmentation occurs in *dmc1 rad51* and *sds rad51* [12,13,45,46]. In *figl1 sds*, the restoration of synapsis is followed by chromosome fragmentation. Both synapsis and fragmentation were absent in the *figl1 sds dmc1* triple mutant (Fig 6D). Thus, DMC1 produces intermediates that promote synapsis and these intermediates in the absence of both FIGL1 and SDS, fail to be repaired. This suggests that DMC1/RAD51 promotes inter-homolog interactions, SDS being a helper in both invasion and repair on the homolog (but not on the sister), while FIGL1 antagonizes inter-homolog interactions. The restoration of DSB repair in *figl1 sds dmc1* compared to *figl1 sds*, and the restoration of DMC1 foci in *fidg sds* compared to *sds*, suggest the possibility that FIGL1 promotes DMC1 turnover, this turnover being required for efficient repair under certain circumstances (e.g. in *sds*). Persistence of DMC1 was also shown to induce DSB repair deficiency in certain contexts in both yeast and *Arabidopsis* [7,9,48].

## Discussion

### FIGL1 and FANCM limit class II COs by distinct mechanisms

Mechanisms that limit COs at meiosis are only starting to be deciphered. Here we identify *FIGL1* as a meiotic anti-CO factor. In *figl1*, extra COs have class II CO characteristics. Indeed, they do not display interference and are not marked by MLH1. Moreover, *MUS81*, which is involved in class II CO formation, becomes essential for DSB repair in *figl1*. Thus, FIGL1 limits class II CO formation, without affecting class I COs, similar to the anti-CO helicase FANCM [24]. However, *FIGL1* and *FANCM* mutations have multiplicative effects on CO formation suggesting that *FIGL1* and *FANCM* mutations fuel the class II CO pathway by two distinct, sequential, mechanisms (see below).

The effect of mutating *fancm* on elevating CO frequency is quite pronounced in inbred lines, but negligible in hybrids. In contrast, increases in CO frequency in *figl1* are similar in inbreds and hybrids. In both inbreds and hybrids, the strongest effect is always observed in the double mutant. Thus, the manipulation of both *FIGL1* and *FANCM* is a promising tool to increase CO formation in plant breeding programs, as COs are one of the principal driving forces in generating new plant varieties but occur at low rates naturally [49–51]. The shrinkage of the anti-CO effect of *FANCM* in hybrids could be caused by the sequence divergence between the parental strains. Ziolkowski *et al*. [36] independently observed a similar result of heterozygosity drastically reducing the *fancm-1* effect in a Col-0/Catania-1 hybrid. They further showed that the large increase in CO frequency in *fancm-1* depends on the homozygous/heterozygous status of the tested interval, independently of the status of the rest of the chromosome, suggesting the heterozygosity acts in *cis* and not in *trans* to prevent COs that arise in *fancm-1*. Ziolkowski and colleagues also draw from their experiments the conclusion that non-interfering (class II) repair is inefficient in heterozygous regions. However, the increase in class II COs in the *figl1* mutant is not affected by the hybrid status. It would therefore indicate that class II COs can occur efficiently in heterozygous regions of the genome, at least in absence of FIGL1. The reason for *fancm* loss of effect in heterozygous regions could arise from mismatches due to heterozygosity that may lead to the production of fewer, or less stable, DNA recombination intermediates [52] on which the FANCM helicase could act [53]. However, the average polymorphism between Col-0 and Ler or Ct-1 is only 1 SNP every ~200pb [54,55] while the gene conversion tracks associated with CO and NCO are estimated to ~400 and less than 50 base pairs, respectively [56,57]. It appears unlikely that so few mismatches, and in many cases none, per recombination intermediate could have such a drastic effect. There may therefore be additional sequence- or non sequence-based mechanisms that impair the anti-CO activity of FANCM in hybrids. The observation that the *figl1* mutation effect on recombination is similar in hybrids than in inbred lines supports the conclusion that FANCM and FIGL1 acts through distinct mechanisms to limit meiotic CO formation.

### A model for the CO-limiting mechanism of FIGL1

Our data show that *FIGL1* regulates the invasion step of meiotic homologous recombination: (i) Mutation of *FIGL1* increases the number of RAD51 foci, (ii) modifies the dynamics of DMC1 and (iii) restores DMC1 foci formation and DMC1-mediated homologous interactions (synapsis) in *sds*. In contrast to *figl1*, *fancm* does not restore homologous interactions in *sds*, supporting the conclusion that *FIGL1* and *FANCM* regulate HR by different mechanisms. One possibility is that *FIGL1* regulates the choice between the homologous and the sister chromatid as repair template. In such a model, the frequency of inter-homologous invasions would be increased at the expense of inter-sister invasions in the *figl1* mutant, leading to more COs. However, several arguments disfavor this simple hypothesis. First, the number of DMC1 and RAD51 foci in wild-type *Arabidopsis* suggests a high number of DSBs, therefore the number of inter-homologue invasions—that cannot be directly estimated currently—probably already outnumbers COs in wild type, making it hard to believe that a further excess would increase CO frequency. Moreover, *MUS81* is essential for completion of repair in the *figl1* background but not in wild type. This suggests that the recombination intermediates produced in the *figl1* mutant differ from those in wild type not simply in their number but in their nature. We therefore propose that FIGL1 prevents the formation of aberrant joint molecules through the regulation of strand invasion intermediates, whose resolution by MUS81 (and possibly other factors) leads to extra-CO formation. FIGL1 could limit the over-extension of the D-loop, and/or prevent the formation of multi-joint molecules by preventing that both ends of the resected DSB interact with different templates and/or by limiting multiple rounds of invasions [58–60].

The multiplicative effect on CO frequency of mutating both *FIGL1* and *FANCM* suggests that they act sequentially. We thus further propose that FIGL1 limits the formation of joints molecules by regulating DMC1-dependant strand invasion and that these joint molecules when formed can then be disrupted by the FANCM helicase. The absence of both *FIGL1* and *FANCM* would lead to a synergistic accumulation of substrates for MUS81, and possibly other factors, accounting for the multiplicative effect on CO frequency.

Alternatively, human FIGL1 was shown to interact with both RAD51 and the KIAA0146/SPIDR protein [32], the latter in turn interacting directly with the BLM helicase [61]. Another, not exclusive, functional hypothesis for the FIGL1 meiotic anti-CO function is that FIGL1 could facilitate the recruitment of the BLM homologues, RECQ4A and RECQ4B, which have been recently shown to also limit meiotic CO in Arabidopsis [62]. It will therefore be interesting to explore the functional relationship between FIGL1 and RECQ4s at meiosis.

FIGL1 is an AAA-ATPase (ATPases Associated with diverse cellular Activities) [63,64], a family of unfoldase proteins [65] involved in the disruption of protein complexes as different as microtubules or chromosome axis components [66,67]. *FIGL1* is the only member of the *FIDGETIN* sub-family to be widely conserved (S1C Fig), contrary to *FIDGETIN* and *FIGL2* that are present only in vertebrates. Arguing for a conserved role of *FIGL1* at meiosis, the mouse *FIGL1* is highly expressed in spermatocytes at meiotic prophase I [33]. Human and *C*. *elegans* FIGL1 orthologs have been shown to form a hexameric ring oligomer, which is the classical conformation for AAA-ATPases [65,67,68]. Several missense mutations identified in our screen fall into the two conserved domains, the AAA-ATPase domain and the VPS4 domain (S1B Fig) [65,69,70] indicating that ATPase activity and oligomerization of FIGL1 are important for its anti-CO activity. Here we show that RAD51 and DMC1 focus formation and/or dynamics are regulated by *FIGL1*. Of interest, the human FIGL1 ortholog has been shown to directly interact with RAD51 in somatic cells [32]. The FRBD domain (the FIGNL1 RAD51 Binding Domain) is necessary for this interaction, and this domain is conserved in *Arabidopsis* FIGL1 (Fig 1 and S1B Fig). An attractive model would be that FIGL1 could directly promote disassembly of the RAD51 and/or DMC1 filaments, preventing unregulated (multi-) strand invasion, and/or the accumulation of DMC1/RAD51 trapped intermediates [71]. However, it is also possible that FIGL1 unfolds another target to regulate CO formation. Such alternative targets could be chromosome axis proteins, e.g. ASY1 or ASY3, which direct recombination towards the homologue [72,73]. This would be reminiscent of the role of another AAA-ATPase that regulates recombination in *S*. *cerevisiae*, Pch2 that targets the ASY1 homologue Hop1 [67].

## Materials and Methods

### Genetic resources

The lines used in this study were: *spo11-1-3* (N646172) [74], *dmc1-3* (N871769)[75], *sds-2* (N806294) [13], *rad51-1* [47], *zip4-*1 (EJD21)[76], *zip4-2* (N568052) [76], *shoc1-1* (N557589)[77], *msh5-*2 (N526553) [78], *mus81-2* (N607515) [22], *fancm-1* [24], *hei10-2* (N514624) [79]. Tetrad analysis lines were: I2ab (FTL1506/FTL1524/FTL965/*qrt1-2*), I3bc (FTL1500/FTL3115/FTL1371/ *qrt1-2*) and I5cd (FTL1143U/FTL1963U/FTL2450L/ *qrt1-2*) from G. Copenhaver [35]. *Atzip4(s)5* (*figl1-1*) was sequenced using Illumina technology (The Genome Analysis Center, Norwich UK). Mutations were identified through the MutDetect pipeline [31].

### Cytological techniques

Meiotic chromosome spreads have been performed as described previously [80]. Immuno-localizations of MLH1 were performed as described in [40], RAD51, DMC1, ASY1 and ZYP1 as in [81,82]. Observations were made using a ZEISS AxioObserver microscope.

### Cloning and transient *FIGL1* expression in *N*. *benthamiana*

The FIGL1 open reading frame was amplified on Col-0 cDNAs with DNA primers (GGGGACAAGTTTGTACAAAAAAGCAGGCTGTAAAGGAATGTGTGGGTCG and GGGGACCACTTTGTACAAGAAAGCTGGGTGAGGCTTAAACTACCAAACTG) and subsequently cloned using the Gateway technology (Invitrogen) into destination vectors pGWB5 and pGWB6 [83] where FIGL1 sequence is in fusion with a GFP protein,. Infiltrations of *Nicotiana benthamiana* leafs with *Agrobacterium tumefaciens* strain C58C1(pMP90) bearing the construction were performed as in [84]

### Fluorescent-Tagged Lines (FTL) tetrad analysis

Tetrad slides were prepared as in [35] and counting was performed through an automated detection of tetrads using a pipeline developed on the Metafer Slide Scanning Platform (http://www.metasystems-international.com/metafer). For each tetrad, classification (A to L) was double checked manually. Genetic sizes of each interval was calculated using the Perkins equation [85]: *D = 100 x (Tetratype frequency + 6 x Non-Parental-Ditype frequency)/2* in cM. (see http://www.molbio.uoregon.edu/~fstahl for details)

The Interference Ratio (IR) was calculated as in [35,86]. For two adjacent intervals I1 and I2, two populations of tetrads are considered: those with at least one CO in I2 and those without any CO in I2. The genetic size of I1 is then calculated for these two populations using the Perkins equation (above), namely D_1_ (I1 with CO in I2) and D_2_ (I1 without a CO in I2). The IR is thus defined as IR = D_1_/D_2_. If the genetic size of I1 is lowered by the presence of a CO in I2, IR<1 and interference is detected. If not, IR is close to 1 and no interference is detected. A Chi-square tests the null hypothesis (H_0_: D_1_ = D_2_.) (S3D Fig).

The coefficient of interference (CoC) was calculated as in [87]. The CoC compares the observed frequency of double CO compared to the expected frequency of double CO without interference. The observed frequency is defined by f_o_(2CO) = frequency of tetrads having at least one CO in I1 and at least one CO in I2 (classes D, E, F, G, J, K, L). The expected frequency is obtained by the product of f_e_(CO_I1_) and f_e_(CO_I2_); where f_e_(CO_I1_) is defined as the frequency of tetrads having at least one CO in I1 (classes C, D E, F, G, I, J, K, L), and f_e_(CO_I2_) as the frequency of tetrads having at least one CO in I2 (classes B, D E, F, G, H, J, K, L). The CoC is thus defined as CoC = f_o_(2CO) / [f_e_(CO_I1_) x f_e_(CO_I2_)]. If the observed frequency of double CO is lower than the expected frequency, CoC<1 and interference is detected. If not, CoC is close to 1 and no interference is detected. A Chi-square tests the null hypothesis (H_0_: f_o_(2CO) = f_e_(CO_I1_) x f_e_(CO_I2_)) (S3D Fig).

### Marker segregation and tetrad analysis in hybrids

Hybrid lines were obtained through the crossing of *fancm-1 figl1-1* double mutant in the Columbia-0 background (bearing the tetrad analysis markers, see above, and the *qrt1-2* mutation) with a *fancm-10 figl1-12* double heterozygous mutant in the Landsberg background bearing the *qrt1-1* mutation [88]. The F1 plants were heterozygous for the tetrad analysis markers and were used to obtain results of Fig 3C. Seeds from the self-pollination of double heterozygote (non-mutant control), *figl1*, *fancm* and *figl1 fancm* plants were sown. DNA extractions were made as in [43] on 21-day-old rosettes.

96 KASPar markers were designed according to their genomic position with an average distance between two markers of 1.5Mb (S2 Table). Genotyping was performed using the KASPAR technology at Plateforme Gentyane, Clermont-Ferrand, France. Genotyping data were analyzed with Fluidigm software (http://www.fluidigm.com). 91 markers gave robust genotyping results and were further kept for analysis on a total of 174 wild type, 223 *figl1*, 174 *fancm* and 166 *figl1 fancm* plants. Results were exported to MapDisto [89]. Genetic maps were computed with Kosambi parameters [90] for each chromosome (in cM, Fig 3B and S3 Fig).

The number of CO per F2 plant was retrieved from the genotyping data. These numbers were then compared between genotypes by a bilateral T-Test (p values are indicated in the main text). To compare recombination along chromosomes, the number of recombinant chromatids was retrieved for each interval (of about 1.5Mb). Super-intervals were obtained by merging adjacent intervals to reach the critical size of ~5Mb. Recombination data from single intervals were then pooled for each super-interval. Chi-square tests were realized to compare wildtype and mutant data. Multiple chi-square test correction was realised using the Benjamini—Hochberg procedure [91]: ** indicates a significant chi-square test with a probability of 5% of false discovery rate, *** indicates a significant chi-square test with a probability of 1% of false discovery rate.

## Supporting Information

S1 Fig The *FIDGETIN-Like1* gene and encoded protein. A: Sequencing of the cDNA revealed a mis-annotation in Genbank: RT-PCR experiments showed that the two *in silico* predicted genes AT3G27120 and AT3G27130 are a single expressed mRNA in vivo (Genbank accession KM055500). B: Alignment with T-COFFEE of FIGL1 proteins from *Arabidopsis thaliana* (At), *Drosophila melanogaster* (Dm), *Homo sapiens* (Sp), *Mus musculus* (Mm), *Caenorhabditis elegans* (Ce) and *Danio rerio* (Dr) showing the FIDGETIN-RAD51-Binding-Domain (FRBD) described in human (Yuan and Chen 2013), the conserved Walker A, Walker B and SRH domains of the ATPase domain (Lupas and Martin 2002; Ogura et al. 2004) as well as the VPS4 domain for oligomerization (Vajjhala et al. 2006). Positions of the mutations found in the different screens are indicated: black stars indicate amino acid changes, red stars indicate mutations to stop codon and green stars indicate mutations affecting splicing site, see also S1 Table. C: Proteins from the FIDGETIN family were identified using literature search and reciprocal BLASTp and PSI-BLAST (http://www.ncbi.nlm.nih.gov/, http://www.*Arabidopsis*.org/ and http://bioinformatics.psb.ugent.be/plaza). Alignments were made by T-COFFEE and subsequent tree building was realized by PhyML on www.phylogeny.fr. Bootstraps values above 0.7 are indicated. Tree rendering was performed on Fig Tree (http://tree.bio.ed.ac.uk/software/figtree) and Adobe Illustrator. Accession numbers for the sequences are: Dm_FIGL1 CG3326 [92], Hs_FIDGNP_060556.2 [93], Hs_FIGL1 NP_001036227.1 [32], Hs_FIGL2 NP_071399.2, Mm_FIDGAAG17289.1 [94], Mm_FIGL1 NP_001156832.1 [33], Mm-FIGL2 NP_001201840.1 [95], Ce_FIGL1 NP_504197 [96], Nb_FIGL1 EOB14776.1, Xl_FIGL1: NP_001086763.1, Dr_FIDG NP_001018411.1, Dr_FIGL1 NP001122223.1, Oc_FIGL1 XP_001421485.1, Trb_FIGL1 XP_844861.1 [97]. D: Over-expression of the FIGL1 protein fused to GFP fluorescent protein infiltrated in *Nicotiana benthamiana* leaves expressing stable histone H2A-RFP fusion protein. H2A-RFP is specifically detected in the nuclei of the cells, as is FIGL1-GFP fusion protein.
(EPS)

S2 Fig Tetrad data analysis. A: Mutation in FIGL1 restores CO frequency in a *zip4* background. Genetic distances (in cM) are measured through tetrad analysis on a pair of intervals on chromosome 5. Error bars: SD. ** indicates p<0.05; *** indicates p<10–3 (Z-Test). B: Tetrad count for all categories (A to L) designed by G. Copenhaver and colleagues [35] for all genotypes and intervals (Columbia-0 inbreds and Col/Ler hybrids) used in this study. C: Genetic distances calculated with Perkins equation [85] from the data of (A). Colors indicate the value of ratio compared to wild type: blue colors indicate ratios below 1 (mutant value below wild-type value), red colors indicate ratios above one (mutant value above wild-type value). D: Interference ratios (IR,[86]) and coefficients of coincidence (CoC [87]), calculated with data from (A) for each pair of interval. When chi-square tests are possible, p-values are given and coloured following their value for the H_0_ hypothesis "IR = 1" or "CoC = 1". The more these measures are inferior to 1, the stronger interference is.
(EPS)

S3 Fig Genome-wide crossover analysis in Col/Ler F1s: wild type, *fancm*, *figl1* and *figl1 fancm* on all five chromosomes. Recombination measurement in cM/Mb obtained using 91 markers on an F2 population. Calculations were made using MapDisto [89]. Each chromosome was then segmented in ~5 Mb super-intervals, and sizes in cM of these super-intervals were compared between each mutant and wild type. Multiple chi-square test correction was realised using the Benjamini—Hochberg procedure [91]: ** indicates a significant chi-square test with a probability of 5% of false discovery rate, *** indicates a significant chi-square test with a probability of 1% of false discovery rate. Blue boxes indicates intervals used for tetrad analysis (FTLs) in hybrids (Fig 2A), yellow boxes indicates heterochromatic centromeric regions, as defined in [43].
(EPS)

S4 Fig The *fancm* effect on crossovers is diminished in F1 hybrids. A. Bivalent frequency in inbreds and hybrids *fancm zmm* mutants. Univalent pairs (red) and bivalents (blue) count of metaphase I, male meiocytes in Columbia (Col), Wassilewskija (Ws) and Landsberg erecta (Ler) backgrounds as well as F1 hybrids Col/Ler or Col/Ws for wild type, *zmm* and *fancm zmm*. Mutation of *FANCM* efficiently suppresses *zmm* lack of bivalent in both Columbia-0 (Col-0) and Landsberg (Ler). In the Col-0/Ler F1 plants *fancm msh4* the frequency of bivalents is not different to F1 *msh4*. It thus appears that the *fancm* mutation is not able to restore CO formation of *msh4* in the Col/Ler hybrid, while it does very efficiently in inbred Col-0 and Ler. We also introgressed the *fancm-1* mutation in a third strain, Wassilewskija (Ws), through four consecutive backcrosses and marker-assisted selection. In Ws, *fancm-1* was able to efficiently restore bivalent formation of the *zip4* mutant. The *fancm-1 zip4* hybrid Col/Ws showed increased bivalent formation compared to *zip4*, but again less efficiently than in the two parental lines. Our data revealed that *FANCM* mutation is efficient at suppressing *zmm* lack of COs in inbred lines (Columbia, Landsberg and Wassilewskija) but less efficient in hybrids (Col-0/Ler and Col-0/Ws). B. Recombination measured in cM along the top arm and centromere of chromosome 1 in wild type and *fancm* mutant in a Columbia-0 inbred. EMS-induced mutations of *fancm-1* and *fancm-2* mutants were used as genotyping markers on 91 F2 plants for each genotype. Calculations and map building were made using MapDisto [89]. The left scale represents the physical maps, with the position of the markers in Mb. Genetic distances in *fancm-1/fancm-2* increased significantly (on average, 117cM compared to 67cM in wild type, T-Test p<10^−6^). The interval spanning the centromere, which has a low recombination frequency in wild type, remains similarly low in *fancm*.
(EPS)

S5 Fig Immuno-localization of ASY1 and ZYP1 in wild type and *fancm-1*. ZYP1 immuno-localization as a marker of the synaptonemal complex, with the chromosome axis protein ASY1 used as a counterstain, at pachytene showing full synapsis. These images showed that the synaptonemal complex track length in *fancm*(125.6μm [n = 32]) is similar to that of wild type (125.5μm [n = 33]).
(TIF)

S6 Fig Immuno-localization of RAD51 and DMC1in wild type and *figl1*. DMC1 and RAD51 double immuno-localization on meiocytes. In wild type 36% RAD51-positive cells were also showing DMC1 foci (n = 59). In *figl1*, 95% of the RAD51-positive cells were also showing DMC1 foci (n = 63), showing that the dynamic of DMC1 with respect to RAD51 is altered in *figl1-1* mutant.
(TIF)

S7 Fig Meiosis in *figl1 sds*, *figl1 sds rad51* and *figl1 sds dmc1*. Chromosome spreads from male meiocytes of each mutant coloured with DAPI. Bottom left zoom for each pachytene image emphasizes the absence of synapsis in *sds*, *rad51*, *figl1 sds rad51*, *dmc1*, *figl1 sds dmc1* and *fancm sds* mutants while revealing synapsis in wild type, *figl1* and *figl1 sds* (see also Fig 6).
(PDF)

S1 Table Nature and position of the mutations used in this study. The positions refer to TAIR10 positions on the Columbia Genome.
(DOCX)

S2 Table Single nucleotide polymorphisms and corresponding KASPar primers used in this study. (DOCX)
