# Correction: AAA-ATPase FIDGETIN-LIKE 1 and Helicase FANCM Antagonize Meiotic Crossovers by Distinct Mechanisms

**DOI:** 10.1371/journal.pgen.1005448

**Published:** 2015-09-17

**Authors:** Chloe Girard, Liudmila Chelysheva, Sandrine Choinard, Nicole Froger, Nicolas Macaisne, Afef Lemhemdi, Julien Mazel, Wayne Crismani, Raphael Mercier

The sixth author's name is spelled incorrectly. The correct name is: Afef Lemhemdi. The correct citation is: Girard C, Chelysheva L, Choinard S, Froger N, Macaisne N, Lemhemdi A, et al. (2015) AAA-ATPase FIDGETIN-LIKE 1 and Helicase FANCM Antagonize Meiotic Crossovers by Distinct Mechanisms. PLoS Genet 11(7): e1005369. doi:10.1371/journal.pgen.1005369
