# Supplementary figures and images for: AAA-ATPase FIDGETIN-LIKE 1 and Helicase FANCM Antagonize Meiotic Crossovers by Distinct Mechanisms
Source: PLoS Genet. 2015 Jul 10;11(7):e1005369. doi: 10.1371/journal.pgen.1005369 (PMC4498898; doi:10.1371/journal.pgen.1005369)

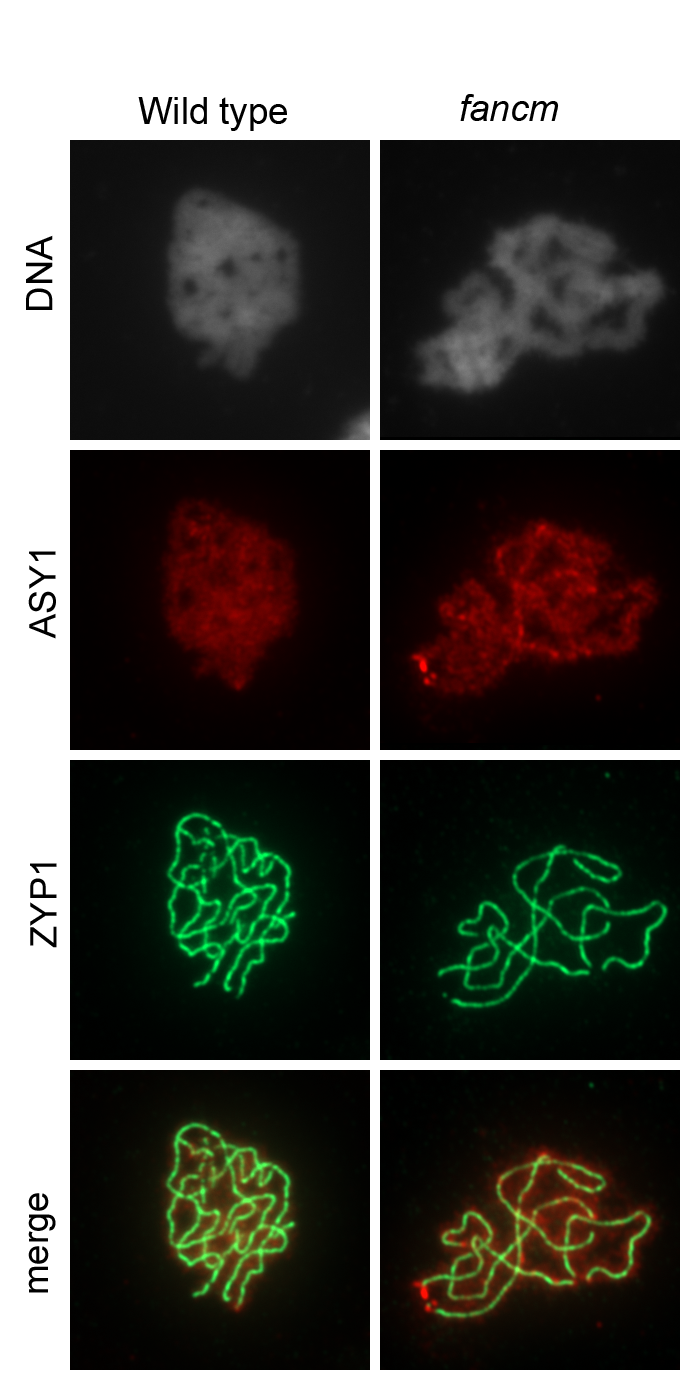

Supplement: S5 Fig — ZYP1 immuno-localization as a marker of the synaptonemal complex, with the chromosome axis protein ASY1 used as a counterstain, at pachytene showing full synapsis. These images showed that the synaptonemal complex track length in fancm(125.6μm [n = 32]) is similar to that of wild type (125.5μm [n = 33]). (TIF) [file pgen.1005369.s005.tif]

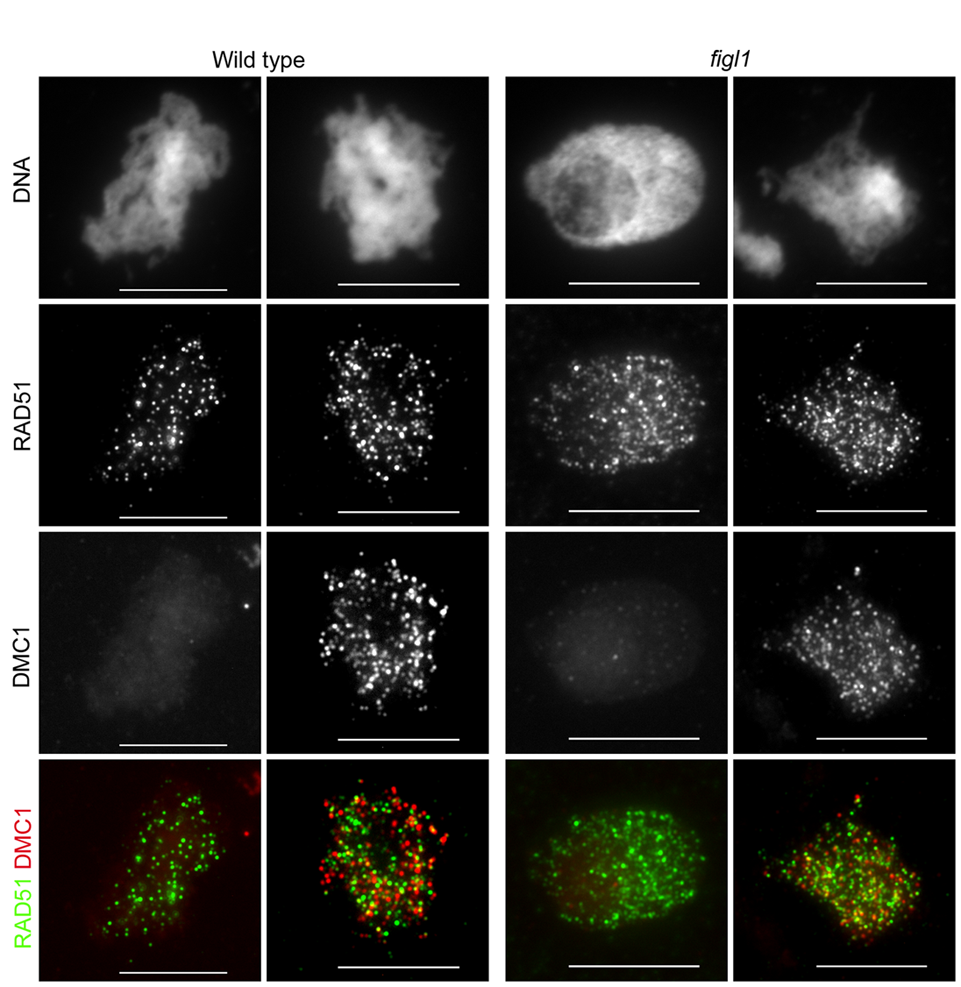

Supplement: S6 Fig — DMC1 and RAD51 double immuno-localization on meiocytes. In wild type 36% RAD51-positive cells were also showing DMC1 foci (n = 59). In figl1, 95% of the RAD51-positive cells were also showing DMC1 foci (n = 63), showing that the dynamic of DMC1 with respect to RAD51 is altered in figl1-1 mutant. (TIF) [file pgen.1005369.s006.tif]

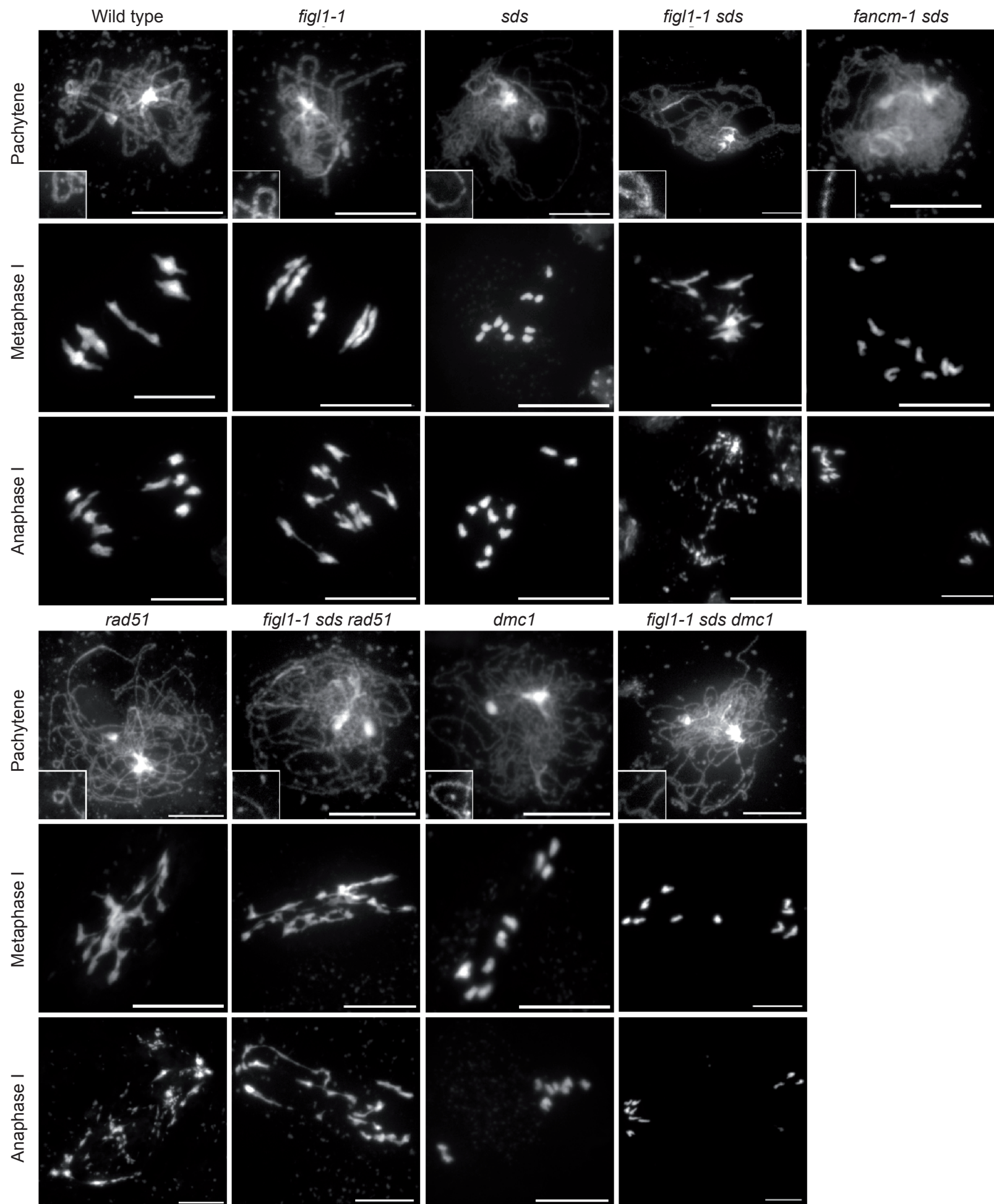

Supplement: S7 Fig — Chromosome spreads from male meiocytes of each mutant coloured with DAPI. Bottom left zoom for each pachytene image emphasizes the absence of synapsis in sds, rad51, figl1 sds rad51, dmc1, figl1 sds dmc1 and fancm sds mutants while revealing synapsis in wild type, figl1 and figl1 sds (see also Fig 6). (PDF) [file pgen.1005369.s007.pdf]
