# Supplementary material for: AAA-ATPase FIDGETIN-LIKE 1 and Helicase FANCM Antagonize Meiotic Crossovers by Distinct Mechanisms
Source: PLoS Genet. 2015 Jul 10;11(7):e1005369. doi: 10.1371/journal.pgen.1005369 (PMC4498898; doi:10.1371/journal.pgen.1005369)
Supplement: S1 Table — The positions refer to TAIR10 positions on the Columbia Genome. (DOCX) [file pgen.1005369.s008.docx]

**Table S1: Nature and position of the mutations used in this study**. The positions refer to TAIR10 positions on the Columbia Genome.

| **Allele** | **Allele in screen** | **Strain** | **Position on  Columbia genome** | **Mutation** | **Change in DNA sequence** | **Change in the protein sequence** | **Position of the amino acid change** |
| --- | --- | --- | --- | --- | --- | --- | --- |
| *figl1-3* | *hei10(s)141* | Col-0 | Chrom3: 10001970 | SNP | C>T | W>STOP | 413 |
| *figl1-5* | *shoc1(s)161* | Col-0 | Chrom3: 10001904 | SNP | C>T | G>D | 435 |
| *figl1-6* | *hei10(s)326* | Col-0 | Chrom3: 10001904 | SNP | C>T | G>D | 435 |
| *figl1-7* | *shoc1(s)123* | Col-0 | Chrom3: 10001812 | SNP | C>T | splicing site | 439 |
| *figl1-1* | *zip4(s)5* | Col-0 | Chrom3: 10001784 | deletion | G>0 | frameshift | 447 |
| *figl1-8* | *msh5(s)652* | Col-0 | Chrom3: 10001772 | SNP | C>T | E>K | 451 |
| *figl1-9* | *hei10(s)235* | Col-0 | Chrom3: 10001422 | SNP | C>T | D>N | 497 |
| *figl1-2* | *zip4(s)4* | Col-0 | Chrom3: 10001128 | SNP | C>T | splicing site | 537 |
| *figl1-11* | *shoc1(s)101* | Col-0 | Chrom3: 10001018 | SNP | G>A | L>F | 545 |
| *figl1-12* | *msh4(s)80* | Ler | Chrom3: 10000840 | SNP | C>T | L>F | 574 |
| *figl1-17* | *msh4(s)65* | Ler | Chrom3: 10000569 | SNP | C>T | A>T | 618 |
| *figl1-14* | *hei10(s)208* | Col-0 | Chrom3: 10000328 | SNP | C>T | E>K | 647 |
| *figl1-15* | *msh5(s)5* | Col-0 | Chrom3: 10000278 | SNP | C>T | W>STOP | 663 |
| *figl1-16* | *msh5(s)647* | Col-0 | Chrom3: 10000279 | SNP | G>A | W>STOP | 663 |
| *fancm-1* | *zip4(s)1* | Col-0 | Chrom1: 13092696 | SNP | G>A | G>D | 510 |
| *fancm-10* | *msh4(s)20* | Ler | Chrom1: 13091152 | SNP | C>T | A>L | 245 |
