# Supplementary material for: AAA-ATPase FIDGETIN-LIKE 1 and Helicase FANCM Antagonize Meiotic Crossovers by Distinct Mechanisms
Source: PLoS Genet. 2015 Jul 10;11(7):e1005369. doi: 10.1371/journal.pgen.1005369 (PMC4498898; doi:10.1371/journal.pgen.1005369)
Supplement: S2 Table — (DOCX) [file pgen.1005369.s009.docx]

**Table S2 : Single nucleotide polymorphisms and corresponding KASPar primers used in this study.**

| Chromosome | position (TAIR10) | marker name | Closest gene | allele col-0 | allele  Ler-0/ WS | Primers | | |
| --- | --- | --- | --- | --- | --- | --- | --- | --- |
|  |  |  |  |  |  | A1 (without tail) | A2 (without tail) | C |
| K1 | 32210 | K1_00032210 | AT1G01050 | T | C | atacgatcaacctgaatgagagaatt | atacgatcaacctgaatgagagaatc | ggttttcttatagcaaaatcaatctcaa |
| K1 | 1282224 | K1_01282224 | AT1G04620 | G | T | ctctgtattaggacaatgcctctgg | ctctgtattaggacaatgcctctgt | aaatgtctctgctcttttgcaccat |
| K1 | 2647801 | K1_02647801 | AT1G08410 | G | A | caccgtattcctgattcatatttacttg | caccgtattcctgattcatatttactta | tcaatttcttgagcatatgcctgtgt |
| K1 | 3938146 | K1_03938146 | AT1G11680 | T | A | cgcagaaaatgggaatttacatt | cgcagaaaatgggaatttacata | ttattcatgtaaaaatccaacataactca |
| K1 | 5254890 | K1_05254890 | AT1G15280 | A | G | actttgtggatgctagttttcagaa | actttgtggatgctagttttcagag | gcaaccaatacgcacgatatctaat |
| K1 | 6612253 | K1_06612253 | AT1G19140 | C | A | cgattgattagggttcacagactgc | cgattgattagggttcacagactga | tgcacttcgtcgattggtggtaata |
| K1 | 7938449 | K1_07938449 | AT1G22490 | T | A | caagttaattataatctatttgctgaatt | caagttaattataatctatttgctgaata | ttgaatcaaaccataaggaggat |
| K1 | 9246484 | K1_09246484 | at1g26740 | T | G | gcatcgcagcgacatgactt | gcatcgcagcgacatgactg | ttgagattgacatcgctaaaacg |
| K1 | 10583364 | K1_10583364 | AT1G30110 | G | A | gaggtcgctaacatttaagttaacacttg | gaggtcgctaacatttaagttaacactta | cccacattagtaaatatatcctctgattcat |
| K1 | 11900533 | K1_11900533 | AT1G32840 | T | C | gttaacacttctttaaggttttaaaatcct | gttaacacttctttaaggttttaaaatccc | ccgtttttaggcataagtaactaaca |
| K1 | 13228440 | K1_13228440 | AT1G35730 | G | A | tgtaatcagaagtaagcggatttag | tgtaatcagaagtaagcggatttaa | cttgcttcaggaagagagaaaaa |
| K1 | 14330125 | K1_14330125 | at1g38176 | A | G | gagcgatgtttgttttatatgcgca | gagcgatgtttgttttatatgcgcg | ttctggtttcttattattcacacga |
| K1 | 15893086 | K1_15893086 | at1g42430 | A | G | accagatatagcttcgttgccaaca | accagatatagcttcgttgccaacg | tgatcaagttgacaatgagaggactg |
| K1 | 17189256 | K1_17189256 | AT1G45688 | A | G | tcttgtgtatagtgatttagttttcgaa | tcttgtgtatagtgatttagttttcgag | ggtgaaataatggatatcaatacg |
| K1 | 18518590 | K1_18518590 | at1g50010 | C | G | accatatcttgttgaatttactgttcattc | accatatcttgttgaatttactgttcattg | cagatcaacaatctctttcccaactga |
| K1 | 19841497 | K1_19841497 | AT1G53200 | T | A | aacatgatgacattatgagccacgt | aacatgatgacattatgagccacga | tctttggctgcattacatccacttg |
| K1 | 21164727 | K1_21164727 | AT1G56500 | G | C | aatcgtcctcaggtacatatgatatag | aatcgtcctcaggtacatatgatatac | aacaacgaatctatcgatgaacaac |
| K1 | 22487959 | K1_22487959 | AT1G61050 | C | T | catctttggaacagagagagtaagaaactc | catctttggaacagagagagtaagaaactt | gacatgagttgatgaatgatgcttcc |
| K1 | 23814880 | K1_23814880 | AT1G64160 | G | C | ctttataatggacacatgcatgg | ctttataatggacacatgcatgc | caattatattgactagtggtgttgtagaaa |
| K1 | 25135161 | K1_25135161 | AT1G67190 | G | A | ccctctcacaaaacttgtggg | ccctctcacaaaacttgtgga | tctagctaaatattgttcacatgatatgtttg |
| K1 | 26449603 | K1_26449603 | AT1G70230 | G | A | cgcttaagaaaagtcttttgaactacg | cgcttaagaaaagtcttttgaactaca | tcctaatatcctataattagatggtgacacg |
| K1 | 27776851 | K1_27776851 | at1g73860 | T | G | gagaagaaaacagagagacggagaaaat | gagaagaaaacagagagacggagaaaag | caaaagatcaaatcaacccacattcc |
| K1 | 29102446 | K1_29102446 | AT1G77450 | G | T | aatctgttatccacaatgcataatg | aatctgttatccacaatgcataatt | gaaatcactaatttggttgtgacga |
| K1 | 30413706 | K1_30413706 | AT1G80950 | C | G | tttgagatgaacgctacaaacgaataac | tttgagatgaacgctacaaacgaataag | gttcttgaaacaatctttgtatctcatca |
| K2 | 68457 | K2_00068457 | AT2G01050 | G | C | tcgacgatgaatccaagtcg | tcgacgatgaatccaagtcc | ggagaccaggtggtgtgtattcaag |
| K2 | 1319899 | K2_01319899 | AT2G04047 | G | A | ccattgctataaaacttaaaccacaaattc | ccattgctataaaacttaaaccacaaattt | tccttcttgttttcttgatttaatcgt |
| K2 | 2589047 | K1_02589047 | at2g06530 | A | G | gtttaagctatgataaggtccattctga | gtttaagctatgataaggtccattctgg | tggatcaatcgaaccaatcttac |
| K2 | 3939602 | K2_03939602 | AT2G10260 | C | T | gtataaaagctggttttacctctttcag | gtataaaagctggttttacctctttcaa | ttgagtctcagattgttgcatttga |
| K2 | 5294466 | K2_05294466 | at2g12900 | T | C | tttttatcatgctttgtgacatttatct | tttttatcatgctttgtgacatttatcc | tgcggctaactctcgatttttca |
| K2 | 6556863 | K2_06556863 | AT2G15110 | T | G | agctgaaggagatcgttcagtgat | agctgaaggagatcgttcagtgag | tgttattcgaactgtcttatgcctca |
| K2 | 7879877 | K2_07879877 | AT2G18130 | A | G | gtgaaccagaacaatcagccatga | gtgaaccagaacaatcagccatgg | ggctccaagatgagctcaagaaagt |
| K2 | 9181810 | K2_09181810 | AT2G21450 | T | G | gattcttgagcaaaagccatctt | gattcttgagcaaaagccatctg | caatcaaagtctatatgtggatgcaga |
| K2 | 10502537 | K2_10502537 | AT2G24690 | A | C | cgaatatgatgatataatcggtcta | cgaatatgatgatataatcggtctc | ggtttcactagctcaaatggtagta |
| K2 | 11817899 | K2_11817899 | AT2G27710 | T | A | ggagtttttagtagccaacaaaagcat | ggagtttttagtagccaacaaaagcaa | gaggtatctcatcaccattcgagtca |
| K2 | 13137294 | K2_13137294 | AT2G30850 | T | C | atgctaagcgagcgctctaccatt | atgctaagcgagcgctctaccatc | aatattttaataaatagtcatcgtggggatg |
| K2 | 14450870 | K2_14450870 | AT2G34220 | C | T | ttcttctcaattcgttcaatgtaaaac | ttcttctcaattcgttcaatgtaaaat | ttctcttgctgcaaacacacatagt |
| K2 | 15726397 | K2_15726397 | AT2G37460 | G | A | gtcaaccgaaaactaaaccgg | gtcaaccgaaaactaaaccga | cagttgtattggctacacctaaacaga |
| K2 | 17071831 | K2_17071831 | AT2G40910 | T | A | ccgcttggttcacgtgctaattt | ccgcttggttcacgtgctaatta | tctgcaaatccgagcatagaatgaa |
| K2 | 18385888 | K2_18385888 | AT2G44530 | C | T | gtaaaacataaacactacttgcaattttc | gtaaaacataaacactacttgcaattttt | tggctccctgagaatatcaaaaa |
| K2 | 19694547 | K2_19694547 | at2g48160 | G | A | aatttagccccttgttgaggtcatag | aatttagccccttgttgaggtcataa | ctggttccaaagtcgaacaaattga |
| K3 | 511 | K3_00000511 | AT3G01015 | A | G | ttgaagcgacatttttttgtgga | ttgaagcgacatttttttgtggg | gcaccgtagatattactcatccggaac |
| K3 | 1303716 | K3_01303716 | AT3G04760 | A | T | caaaacgtcttccgggaccaa | caaaacgtcttccgggaccat | attgacagtccatagctgcaactgc |
| K3 | 2599949 | K3_02599949 | at3g08550 | T | C | ctaaccggtaatgactcaacacgt | ctaaccggtaatgactcaacacgc | ttttggaatcctagattgtgtcatcc |
| K3 | 3909888 | K3_03909888 | AT3G12260 | C | A | cacataaactacttgcacaaattctac | cacataaactacttgcacaaattctaa | gtgttgttgaaagctgaaagc |
| K3 | 5247561 | K3_05247561 | at3g15518 | G | A | gaaaaagaaagaaagattctgtataaacagag | gaaaaagaaagaaagattctgtataaacagaa | tgctgttcttttcatcttaatcgct |
| K3 | 6516618 | K3_06516618 | AT3G18895 | C | A | ccatgaagatgattttatccc | ccatgaagatgattttatcca | caaaacctcatttaatttgtgtagaa |
| K3 | 7819492 | K3_07819492 | AT3G22160 | G | T | cgtttctttgaagatcgttgcg | cgtttctttgaagatcgttgct | gcagagaaagatttgccttcacaat |
| K3 | 9133998 | K3_09133998 | at3g25070 | T | C | gtacggcctgagtgatttggtaact | gtacggcctgagtgatttggtaacc | tgaacattagaaacaacaaacttggaggt |
| K3 | 10424554 | K3_10424554 | AT3G28030 | T | C | agaagggagatatggttcagaat | agaagggagatatggttcagaac | aaagcttacaaatccttcgaaaa |
| K3 | 11725238 | K3_11725238 | AT3G29800 | T | C | agatatggcccggttttggcct | agatatggcccggttttggccc | ccacatatatttcataatatgggcttcg |
| K3 | 12784540 | K3_12784540 | at3g31425 | G | T | atatatacccatataatttctaagtatgcag | atatatacccatataatttctaagtatgcat | tacattctttttcgttgccatgtga |
| K3 | 14325353 | K3_14325353 | at3g42180 | A | T | cttggtccgcccatcacgta | cttggtccgcccatcacgtt | tggttcacgatggaccggtaa |
| K3 | 15640050 | K3_15640050 | AT3G43730 | A | C | gagaacaaatcagtcatgcatttaca | gagaacaaatcagtcatgcatttacc | tttagctgaaagtctttgtgattga |
| K3 | 16942558 | K3_16942558 | AT3G46130 | T | A | tcatagaggagtgtgttggtgt | tcatagaggagtgtgttggtga | actgattcttatagtggagccagat |
| K3 | 18251996 | K3_18251996 | at3g49220 | G | T | ttcaactcaggaaccttctcatg | ttcaactcaggaaccttctcatt | gcatgagcaagaaaaagttggtcaaa |
| K3 | 19559589 | K3_19559589 | at3g52770 | C | A | tctttccccatctcatgtccc | tctttccccatctcatgtcca | tggtggagtgtaatatttgaactaaaccaag |
| K3 | 20845079 | K3_20845079 | AT3G56180 | T | C | catacatataaaaatgactctttcttgaaat | catacatataaaaatgactctttcttgaaac | acttgttcttcaacgcaagtcc |
| K3 | 22155886 | K3_22155886 | AT3G59990 | A | G | gtacaatctcgacaacagtatgagata | gtacaatctcgacaacagtatgagatg | ctactgattcagatcctctgttttg |
| K3 | 23456808 | K3_23456808 | AT3G63530 | T | G | gagttcctgagacaaccccct | gagttcctgagacaacccccg | aggaattagtagagctgggggaagc |
| K4 | 1470 | K4_00001470 | at4g00005 | T | G | gtgggcgggaagagagaaactgat | gtgggcgggaagagagaaactgag | atttcagggtgaattacgtctgcat |
| K4 | 1327356 | K4_01327356 | AT4G03000 | T | C | gctcgcgggtaaaagcgctt | gctcgcgggtaaaagcgctc | gcagtccaaagagataaaaccaaagca |
| K4 | 2753291 | K4_02753291 | AT4G05430 | G | T | attggtcatcaaatttaaagatcg | attggtcatcaaatttaaagatct | tcacataaccataagaacaagcctta |
| K4 | 4013047 | K4_04013047 | AT4G06718 | T | G | ggattgagacacagtttgaccatat | ggattgagacacagtttgaccataG | ttttgcttagaaacagtaaacagctttg |
| K4 | 5316459 | K4_05316459 | at4g08390 | T | G | ccagagtggctgaagtttgataattct | ccagagtggctgaagtttgataattcg | agtgtgaagatagagttaaagttctga |
| K4 | 6620178 | K4_06620178 | AT4G10760 | G | T | acaacaatatcatatgacagttaaaagg | acaacaatatcatatgacagttaaaagt | tcgagttattcgcccgtatg |
| K4 | 7967385 | K4_07967385 | AT4G13720 | T | C | tcgaggtttaatacaatgctttctgggatat | tcgaggtttaatacaatgctttctgggatac | atgggatccagtgtttcaacctgat |
| K4 | 9292483 | K4_09292483 | AT4G16480 | A | T | tttgagtcaaggacgtttgca | tttgagtcaaggacgtttgct | cctcgagggaggagcattttc |
| K4 | 10620078 | K4_10620078 | AT4G19490 | T | A | ttaacagattggtggaaggttgggt | ttaacagattggtggaaggttggga | aaaggctttggcttgtgattgcag |
| K4 | 11946695 | K4_11946695 | AT4G22740 | T | C | taagaatcaaaaattgtagccatgt | taagaatcaaaaattgtagccatgc | tgggcatgcaaagttctaaatt |
| K4 | 13262843 | K4_13262843 | at4g26190 | A | T | gacacattcaatgcataagcctattcaa | gacacattcaatgcataagcctattcat | aaggccttgtgtaatcctgtaagtg |
| K4 | 14609495 | K4_14609495 | AT4G29890 | T | C | gggagaaagtcttgttttgtttgcct | gggagaaagtcttgttttgtttgccc | cagcgcataatcagttgagttacgg |
| K4 | 15938582 | K4_15938582 | AT4G33030 | T | G | gtatctacgattttgtctctgttgttt | gtatctacgattttgtctctgttgttg | acttttcctcattggtgctattttc |
| K4 | 17258002 | K4_17258002 | AT4G36580 | G | C | cggcggcgatagctgctgcg | cggcggcgatagctgctgcc | atcggcgtaagcacggttctgagaca |
| K4 | 18584386 | K4_18584386 | AT4G40100 | T | C | ggttaacccttcgataagcacagtt | ggttaacccttcgataagcacagtc | tcgaccgagattacctggatgtact |
| K5 | 257 | K5_00000257 | AT5G01010 | T | C | gattatcctataattatgttttgcggcttt | gattatcctataattatgttttgcggcttc | tccctgactcgaccactaaacaaca |
| K5 | 1283107 | K5_01283107 | AT5G04500 | T | C | gtcccctaaattaaatgcgtatt | gtcccctaaattaaatgcgtatc | gtaagtactacaaattaataagtacattggat |
| K5 | 2571017 | K5_02571017 | AT5G08010 | T | C | cgtctgcttggagttatcaattcat | cgtctgcttggagttatcaattcac | tcctctgactctcttgtaaggcattctc |
| K5 | 3851004 | K5_03851004 | AT5G11940 | T | G | aactttttcaggttgaagcaaagt | aactttttcaggttgaagcaaagg | ataataggaagaccttcggaaact |
| K5 | 5134094 | K5_05134094 | at5g15740 | T | G | cttgcactaaccaatatataagaggagct | cttgcactaaccaatatataagaggagcg | ttcaccaacttattcaatatgctcca |
| K5 | 6423684 | K5_06423684 | AT5G19140 | T | A | cccaaagtttctaccgtcggt | cccaaagtttctaccgtcgga | cgaaatttaaaggctcgacgatgat |
| K5 | 7708344 | K5_07708344 | AT5G23010 | A | T | ccatccaaggccatgatagtcttaaa | ccatccaaggccatgatagtcttaat | aacataatttgcgtatgatatttgagttga |
| K5 | 8990695 | K5_08990695 | AT5G25810 | C | G | gagcaatgaatcacaatgcatgc | gagcaatgaatcacaatgcatgg | gcatttgacaactaagttttgtatgatcg |
| K5 | 10276042 | K5_10276042 | AT5G28288 | C | T | taatcccatgcaaagcacaagtac | taatcccatgcaaagcacaagtat | ttttgtttttggtctcagcgtttgt |
| K5 | 11543254 | K5_11543254 | AT5G31412 | A | T | ccgtcaggtccaaactgtcaca | ccgtcaggtccaaactgtcact | tgccattgataatgatgcttttagga |
| K5 | 12892869 | K5_12892869 | at5g34450 | T | G | cattttgaacactctatatagattcactgat | cattttgaacactctatatagattcactgag | tcgattcgatgtccctgagatc |
| K5 | 14136468 | K5_14136468 | at5g35995 | G | C | aggacaattgaagggtcctgcag | aggacaattgaagggtcctgcac | tctctaagcgtctacgggcttgact |
| K5 | 15415102 | K5_15415102 | AT5G38500 | C | G | ctcaaaggagaataggaattacctctgac | ctcaaaggagaataggaattacctctgag | tccaggaacgatcttaaacattctga |
| K5 | 16711286 | K5_16711286 | AT5G41761 | A | C | gtgcttgcatctacatgatcca | gtgcttgcatctacatgatccc | gggagatgtgtttatataggcaaaacaa |
| K5 | 17981293 | K5_17981293 | at5g44582 | G | A | tcgccgtgttgaaattagatg | tcgccgtgttgaaattagata | ttaatcaatcgcctacctaataaaaa |
| K5 | 19267229 | K5_19267229 | AT5G47490 | T | A | caacttgggcaacaggtttgtt | caacttgggcaacaggtttgta | ccaagtcagtcaaccaagcactaca |
| K5 | 20543859 | K5_20543859 | AT5G50450 | G | A | cggaaccacgcttaaatcagttaagg | cggaaccacgcttaaatcagttaaga | cgcgtgttctctacgctcttacctc |
| K5 | 21843660 | K5_21843660 | AT5G53790 | A | G | cgcaatcaatcacaggtagaagaaa | cgcaatcaatcacaggtagaagaag | gttgaacaacatgtatggggtttgc |
| K5 | 23217719 | K5_23217719 | at5G57320 | G | C | gacctatttaacatagtcttattgatctttg | gacctatttaacatagtcttattgatctttc | gatgccctctttgtgtgtttcttga |
| K5 | 24404922 | K5_24404922 | AT5G60700 | C | T | agacatggtgaaacggtagcgtc | agacatggtgaaacggtagcgtt | aggaaaacactttgtgctcagcttg |
| K5 | 25702570 | K5_25702570 | AT5G64260 | C | A | gcgtggttcgtacgctacgtc | gcgtggttcgtacgctacgta | cgggtgcactcgagtagttagttcag |
| K5 | 26974816 | K5_26974816 | AT5G67640 | C | G | gcatcaaattcaaattgtcgaac | gcatcaaattcaaattgtcgaag | catgtgagtccatttttgtgagttt |
